# Supplementary material for: Emerging heterogeneous compartments by viruses in single bacterial cells
Source: Nat Commun. 2020 Jul 30;11:3813. doi: 10.1038/s41467-020-17515-8 (PMC7393140; doi:10.1038/s41467-020-17515-8)
Supplement: Supplementary file 1 — Supplementary Information [file 41467_2020_17515_MOESM1_ESM.pdf]

Supplementary Information

for

Emerging Heterogeneous Compartments by Viruses in Single Bacterial Cells

Jimmy T. Trinh, Qiuyan Shao, Jingwen Guan, and Lanying Zeng

This supplementary file includes:

Supplementary Discussion

Supplementary Figures 1-15

Supplementary Tables 1-3

Supplementary References

Supplementary Discussion

Detailed explanation of the *tetO*/TetR reporter

The basic functions of the Tet reporter are described in the main text, where a fluorescent TetR-FP fusion protein binds to *tetO* sites on phage DNA for labeling. As for explicating details, the TetR-mCherry protein is constitutively expressed from a plasmid in all of our cells. This makes the cells become populated with fluorescent proteins whether or there is phage DNA or not. TetR will bind to a single phage DNA molecule with a 24x *tetO* array, but we cannot detect a single phage DNA under our imaging conditions. It is unclear how many phage DNAs are required to exist in a single location before a discernible signal presents itself under our conditions. Because of this behavior, this TetR reporter is truly a localization reporter of phage DNA clusters. For example, if 10 phage DNAs were even distributed throughout the cell, it should not be visible with our method, but if those 10 DNAs were in the same approximate location, there would be a cluster in the image.

In the early time points of our representative images, cells are filled with TetR signal (for example, 40 min in the Supplementary Fig. 2c), and the channel is labeled as Replicated DNA or similar, but that signal represents free TetR, not phage DNA at those time points. In subsequent time points, there are smaller areas of stronger TetR signal, contrasted with dimmer signals. Only these local maxima represent phage DNA, the dimmer signals do not. To properly evaluate similar images, only the local maxima should be considered when looking for phage DNA locations. Local maxima can only form when there are sufficient binding sites for TetR, otherwise the entire TetR population will be diffuse. The only way TetR will abrogate its diffusive localization is if it is bound, so the location of phage DNA, if it is organized will force the TetR signal to stop filling the cell. In the local maxima, there are both the diffusing TetR and the bound TetR, so the signal is stronger in that specific area. This behavior is not physically possible without the clustering of phage DNA. The kymographs should also be evaluated to understand phage DNA replication. At early time points, the TetR signals are spread throughout the kymograph, which is not true of the actual phage DNA, since it would be too early for extensive phage DNA replication. Later, there should be a clear rearrangement of the signal pattern into dimmer and brighter areas. The bright areas after this time represent phage DNA.

All images are graphical representations of quantitative data. There are many ways to process these data into different-looking images. For TetR images, sometimes a single contrast setting was chosen, such that when an image appears brighter, it represents a quantitative increase in the signal. Thus, peaks in the data are equivalent to clusters in the images. A setting was chosen for the presented images that included background signal at early time points in particular, which obscures the small TetR clusters somewhat (Fig. 3 or Supplementary Fig. 2 images). It would be simple to choose a different contrast to show small TetR clusters as foci (for example in Fig. 1e, 80 min), but that same contrast would make the TetR clusters disappear at later time points because the signals become weaker over time as they spread across the cell and photobleach (Fig. 1e, 180 min for example would be completely black). The alternative would be to pick a different contrast for each time point, which we decided to do for the images

in Figure 1, but not Figure 3. The fixed-contrast images were included to allow comparison (Supplementary Fig. 2c,e).

Data analysis comments

For spatial analysis of cell data, we normalize positions on every cell, regardless of size. Absolute distances/sizes are retained and calculated using our microscope's calibration for specific figures (64 nm/pixel). The light diffraction limit is ~250 nm, so we cannot conclude that distances/sizes around these values are correct, but the values are calculated based on pixels and are stored in our dataset.

For different heat map figures, the data are normalized in one of two ways, to the population maximum or within each cell. When normalizing to the population maximum, the data show differences between bright cells versus dim cells and show how fluorescence changes over time, such as in the kymographs. Normalizing within each cell removes the effect of particularly bright cells in the population, such that a peak location can be easily visualized regardless of the relative brightness of the cell to the population.

We draw cell masks based on phase contrast, and cellular fluorescence does not reach the extremities of the cells under phase contrast. In effect, this does not allow heat maps to be flat. This also biases the spatial preference of fluorescence away from the poles, such that signals can only approach, but generally not reach, the cell poles.

Rationale for lysogen induction experiment

The rationale behind the induction experiments is that the induction process produces multiple intracellular phactories more consistently. We mention "specifically" in the main text because phage infections have more elements of randomness affecting the separation of phage DNAs. An MOI > 1 would facilitate multiple phactories during infection, but phage adsorption for any given cell follows the Poisson distribution[1], so it is not possible to guarantee that all cells have multiple phages. Even when multiple phages adsorb to the cell, there are preferred positions for phage adsorption[2], such that multiple phages may land in the same vicinity and eject their DNA correspondingly, resulting in non-separation of phage DNAs in the cell. This assumption takes into account previous reports that phage DNAs tend to behave subdiffusively, remaining near their translocation site[3,4]. Even when multiple phages attach to different areas in the cell, there is an intrinsic failure rate for phage DNA ejection[5], so only one DNA may enter the cell even when multiple adsorb. Furthermore, even if phages attach to different areas on the cell, and eject their DNA successfully, the subdiffusive behavior of phage DNA is not absolute, as phage DNAs can move throughout the cell[4,6], allowing once-separated DNAs to coalesce before replication.

Lysogenic induction mitigates the above obstacles to separating phage DNAs in the cell. We perform the induction when the lysogens are in the exponential phase, so all growing cells should have multiple nucleoids which are organized to different parts of the cell[7], therefore multiple prophages are inherently separated before lytic development. As the induction process

progresses, the phage DNAs will excise from the nucleoid, and may be free to diffuse in the cell and coalesce, but this method at least controls the starting point of multiple phage DNAs in all cells.

Choosing the contrast of fluorescence images

For fluorescence images, we must choose contrast settings for representative images. We choose a single contrast setting for any given figure panel, unless indicated otherwise. This allows time-lapse images to be compared to each other. We generally do not allow the background-level fluorescence to be visible, because then, the actual cellular signals would be oversaturated, and the images would yield little useful information. This is the same reason why we choose a contrast to allow only the foci to be clearly visible. Signals do exist throughout the remaining inside and outside of the cell, but they are not as bright as those in clusters.

Comments on lysogeny

There are key differences between lytic and lysogenic development, which we examine with our reporters. The rationale behind the focus on lytic cells is because lysogeny does not depend on sustained DNA replication, as mutants lacking phage DNA replication can lysogenize, although they are compromised[8]. Here are brief observations about lysogeny from our dataset. In lysogeny-deciding cells, yellow signal accrues, and cells do not lyse (Supplementary Fig. 3d; Movie 2). The mKO2 protein for the lysogenic reporter is shared with the SeqA reporter, but because lysogeny occurs late, we are able to observe the initial infecting DNAs and lysogenic outcomes without conflict. We find that DnaB foci are neither as prevalent nor long-lasting during lysogeny (Supplementary Fig. 3b). Consequently, lysogenic cells lack red clusters, although low levels of DNA replication should be occurring. This suggests that the reduced DnaB recruitment and DNA replication for lysogenic cells is below our detection limit.

Comments on distance measurements of reporters over time

The rationale behind using negative time points in Supplementary Fig. 4a-d was to show how locations of one reporter predict the appearance of another. If the difference between locations decreases as time approaches zero, it suggests that the preceding reporter is ceasing its movement in the location of the following reporter. These observations are also because of our limits of detection for TetR and DnaB clusters. When we observe no organization of DnaB in our uninfected control, we confirm that we cannot detect a single functional DnaB unit, a hexamer. This indicates that phage DNA can undergo initial DNA replication, with the required DnaB, before we can detect either the DnaB clusters or TetR clusters.

Regarding the observation that the SeqA-DnaB distance actually increases over time, this is likely due to the original hemi-methylated DNA strands shuffling about the multiple phage DNAs that exist over time. After acting as the original template, the parent strands of the infecting phage DNA separate during replication, and this process repeats itself with more DNA replication. Therefore, the methylated, SeqA-bound strands can reasonably exist anywhere within the TetR cluster. It is unclear if the DnaB is preferentially bound to a specific sister phage

genome after replication or not, but the data suggest that DnaB need not remain on the original phage DNA strands.

Regarding the trend of the TetR-DnaB plot, the reason that most of the data are exactly zero is because of our calculation method. Whereas the SeqA-DnaB distance is calculated as the pixel distance converted to nm, the TetR-DnaB distance is calculated as the pixel distance between the designated DnaB spot and the boundary of the TetR cluster, unless the DnaB location overlaps with the inside of the TetR boundaries, which is then designated as zero distance. Since DnaB is originally attached to SeqA, and SeqA remains in the TetR cluster, it is very likely for TetR-DnaB to be equal to zero, making the graph appear as it is. This is also why the co-localization plot is shown, because the very small measured distances are lower than our resolution limit.

Explanation of fluorescence scatter plots over time

For the live-cell infection and lysogenic induction experiments, the fluorescence data were plotted in Supplementary Fig. 7, 8, and 11. By plotting the fraction of fluorescence in the designated cluster for each cell, we analyze the spatial preference of different reporters. The line with a slope = 1 represents the behavior of a reporter with perfectly diffuse fluorescence, so points above the line suggest a preference for the reporter being within a designated cluster. We stated that these plots are indicative of heterogeneity for different processes in space. We show this using standard deviations of signals for different sizes of clusters because a specific size of a TetR cluster does not represent a homogeneous state of development. Variability in signals represents different levels of DNA and packaging occurring over time.

Rationale for *attB* experiment

When analyzing how TetR clusters grew, we observed that the clusters never fill the whole cell. We confirm the negative correlation between cluster growth vs. size, which is the predicted behavior if the cluster is being prevented from filling the cell. From this, we reason that there should be an opposing force slowing the growth of phage DNA, and that the *E. coli* nucleoid would be a reasonable subcellular object that could achieve this effect.

Explanation of *attB* expansion analysis

The rationale behind our analysis for Supplementary Fig. 10e-f was to show how TetR clusters push *attB* in general, not just in our designated subclass of “push.” By calculating the *attB*-TetR boundary distance, we differentiate between different behaviors of the nucleoid marker with replicating phage DNA. In the scenario where *attB* could freely enter the cluster, we consider this situation as a single arbitrary location on the cell, because the TetR boundary would simply pass through an arbitrary location. In this case, the initial distance for the population would be non-zero. The expectation is that the distance from the cluster edge would first decrease as TetR grows in size and go to zero as TetR approaches the fixed location. Then, the absolute distance would increase as TetR expands to fill cell and overtakes the fixed location. In another scenario, where there is a barrier both impermeable to phage DNA and

immune to being pushed by TetR expansion, we consider this situation as an arbitrary location on the cell that prevents the TetR boundary from expanding beyond the location. In this case, the initial distance for the population would be non-zero and would approach zero as the TetR cluster grew. The difference between this scenario and the above one is that there would be a dearth of data points for larger TetR sizes because the barrier would be unmoved by TetR. Neither of these described scenarios matches the trend of our experimental data. In the experimental analysis, following the initial non-zero distribution, the distance shrinks and remains small even as TetR fills the cell. This suggests that after TetR grows and meets the location of *attB*, it does not overtake *attB* freely, because the absolute distance must increase if the TetR could overtake *attB*. Thus, the nucleoid acts as a barrier. Examining the bin sizes for different TetR sizes, we see that each bin, outside of the extremes, is comparable, suggesting that the nucleoid does not particularly resist the expansion of TetR. Thus, phage DNA moves its barrier, because an immovable barrier would force the TetR size to remain small. Therefore, the trend of the data supports our working model.

Explanation of DAPI-free phage DNA

It is unintuitive to suggest that phage DNA can exist in DAPI-free locations, given that DAPI stains DNA. As mentioned in the main text, *E. coli* DNA is much larger than lambda DNA (~100 fold), which means that DAPI will stain more bacterial DNA, resulting in more signal. It is more correct to state that phage DNA, at early time points before extensive DNA replication, exists in local DAPI minima, but the point is that phage DNA can appear to be relatively unstained by DAPI. Since we must choose a single contrast setting for any given image, there actually is fluorescence in the DAPI-free locations, but it is not visible under our settings.

Rationale for difference maps

We calculate our difference maps as a non-absolute-value differences. The effect of this is that the difference map specifically emphasizes where peak signals in one map anti-correlate with low signals in the other. The stated observations are that differences between *attB* and DAPI are small over time, and differences between *attB* and phage DNA are large over time. Differences between phage DNA signal and DAPI decrease over time. So even though phage DNA signal becomes DAPI signal over time, phage DNA signal does not become *attB* signal, even though *attB* signal is always in the location of DAPI signal. Phage DNA DAPI is therefore different than *attB* DAPI, which we interpret as phage DNA pushing the nucleoid away in accordance with our live-cell data. We note here that the contrast for the difference maps is equivalent for all of difference maps for Fig. 4 and Supplementary Fig. 13.

Explanation of RNA FISH signal plots

The rationale behind the analysis in Supplementary Fig. 14b-c, e-f was to show how the locations of different signals correlate throughout the dataset. For pR (Supplementary Fig. 14b), the brightest pR locations correspond to off-peak DAPI signals, which is also represented in Supplementary Fig. 14d, showing that the brightest median pR signals occupy the dimmer DAPI signals, consistent with the conclusion of anti-correlation of these signals.

Supplementary Fig. 14e-f are meant to be compared. The stated observation is that the brightest pR' locations also contain very strong pR signals. The brightest pRE locations contain stronger pR signals than weaker pRE locations, but not as strong pR signals as the brightest pR' locations. That the pRE/pR correlation is weaker than the pR/pR' correlation suggests that pRE foci are generally offset from the pR foci, despite being within their general vicinity. Examples of this pR/pRE offset can be observed in Fig. 5f-g, in contrast to the pR/pR' co-localization.

Explanation of within-cell versus between-cell analysis

The design of this analysis, results shown in Supplementary Fig. 14g, was to take an unbiased look at the dataset and characterize how much different cell locations vary as compared to different cells in the same dataset. One broad way to understand the analysis is to consider each cell as two separate cells, and the analysis would then compare the within-cell pairs with random whole-cell pairs. We used the data from the RNA FISH experiment 15 min after infection, without further treatment. The pR mRNA and DAPI signals were chosen for this analysis because pR is typically understood to be the transcript that is influencing the downstream decision because it contains the major decision-making proteins. In other words, different levels of pR are predictors of CII and Q levels which bias the decision. DAPI is an internal control for the analysis. In these normally growing cells, there are typically either 2 or 4 nucleoids, and they are fairly evenly spaced on both cell halves, so DAPI in one half of the cell will be similar to the other half on average. The average DAPI level between cells is also expected to be similar, but not as similar as within the same cell due to cell-to-cell staining differences.

The results from the within-cell analysis were generated by obtaining a ratio of the average signal between cell halves for each cell and reported as the median of the distributions of the ratios for pR and DAPI. The results from the between-cell analysis were generated by bootstrapping pairs of cells (n = total cells in FISH dataset) 1000 times. Each bootstrap resulted in a distribution and median, and the median from each of the 1000 runs was plotted as a distribution for pR and DAPI, which was compared to the within-cell median. The within-cell DAPI was less variable than the between-cell DAPI, and the between-cell DAPI distribution was tight, as expected. This indicates that the analysis captures the data in an expected manner, which helps validate the data when looking at pR.

Testing the DnaB-mTurquoise2 constructs

Experiments were done to verify the insertion and growth of strains with the *dnaB-mTurquoise2* allele (Supplementary Fig. 2). The DnaB-mTurquoise2 strains (LZ1511 and LZ1555) were generated by first constructing a high-copy number plasmid with the full *dnaB* gene with a C-terminal fusion to mTurquoise2, a Cm resistance cassette and a homology region downstream of the *dnaB* gene. As a result of this, we noted leaky expression of the fluorescent fusion, which informs us that the DnaB-mTurquoise2 construct was being produced and notably, that this construct does not impose a dominant negative effect on cell growth. Using red

recombination, we produced the genomic construct. The construct was initially verified by selection using Cm and microscopy, which showed a distinct fluorescent phenotype.

The DnaB-mTurquoise2 reporter strain (LZ1555) was verified with PCR using different sets of primers. There was some concern that the *dnaB* gene could be duplicated during recombination, which would create a copy of *dnaB* without the fluorescent fusion, and this would often arise nearby the site of recombination due to an unequal crossover event. We used a single forward primer within the *dnaB* gene (*f-mid-dnab*) paired with three reverse primers in the Cm resistance cassette (*r-cmr*, pair1), the downstream homology region we inserted on the plasmid (*r-h-dnab*, pair2), and a further downstream region on the *E. coli* genome (*r-down-dnab*, pair3), which was not inserted on the plasmid. The rationale for this was that an adjacent *dnaB* duplication could be detected by the presence of two PCR bands by all the three pairs. For the native DnaB strain (LZ1663), no band would be produced by the pair1 and one band would be produced by pair2 (~500 bp) and pair3 (~800 bp), which would be smaller compared to LZ1555 due to not having the fluorescent protein or resistance cassette. A proper insertion of the construct would produce single bands by pair1 (~1600 bp), pair2 (~2200 bp), and pair3 (~2600 bp). The PCR results showed properly sized single bands by all pairs in LZ1555 (Supplementary Fig. 2f), and sequences for those bands were accurate. When the control strain LZ1663 was tested, there were smaller bands corresponding to pair2 and pair3 only. These results coupled with the apparent non-toxic behavior of the DnaB-mTurquoise2 protein, as indicted by the recombination plasmid, suggest that the constructs were inserted as intended without pressure for the cell to duplicate *dnaB*.

We also tested the behavior of the DnaB-mTurquoise2 construct with respect to cell and phage growth, because the fact that the DnaB-mTurquoise2 construct can form colonies might still belie other negative effects on bacterial and phage biology. When comparing growth curves of the parental strain MG1655 and LZ1511 (MG1655, *dnaB-mTurquoise2*), we found that both strains had similar doubling times in LB and M9 media (Supplementary Fig. 2g). This indicates that there is no negative effect imposed by the DnaB-mTurquoise2 fusion itself, which is important to note. We performed a lysogen induction of the same phage with or without the DnaB-mTurquoise2 construct, finding that both strains lysed at the same time and resulted in similar titers for the lysate (Supplementary Fig. 2h). This indicates that lambda growth is also not negatively affected by the DnaB-mTurquoise2 construct. We also examined the size of cells with and without the DnaB-mTurquoise2 construct and found that in the presence of DnaB-mTurquoise2, cells were slightly longer, and all cells had similarly normal and tight distributions (Supplementary Fig. 2j). These results suggest that there is a minor effect of DnaB-mTurquoise2 on growth, but that any effect appears to be small and consistent across the population. For cells with the *dnaB-mTurquoise2* allele, there are neither secondary peaks on the distributions nor are there wide distributions to indicate that the DnaB-mTurquoise2 fusion is heterogeneously expressed to create a sick population or affect different cells in markedly different manners. Altogether, the results indicate that the DnaB-mTurquoise2 construct is expressed as it should be and does not greatly compromise the function of DnaB to inhibit cell viability. We therefore interpret the localization results of DnaB to be properly informative of natural cell behavior.

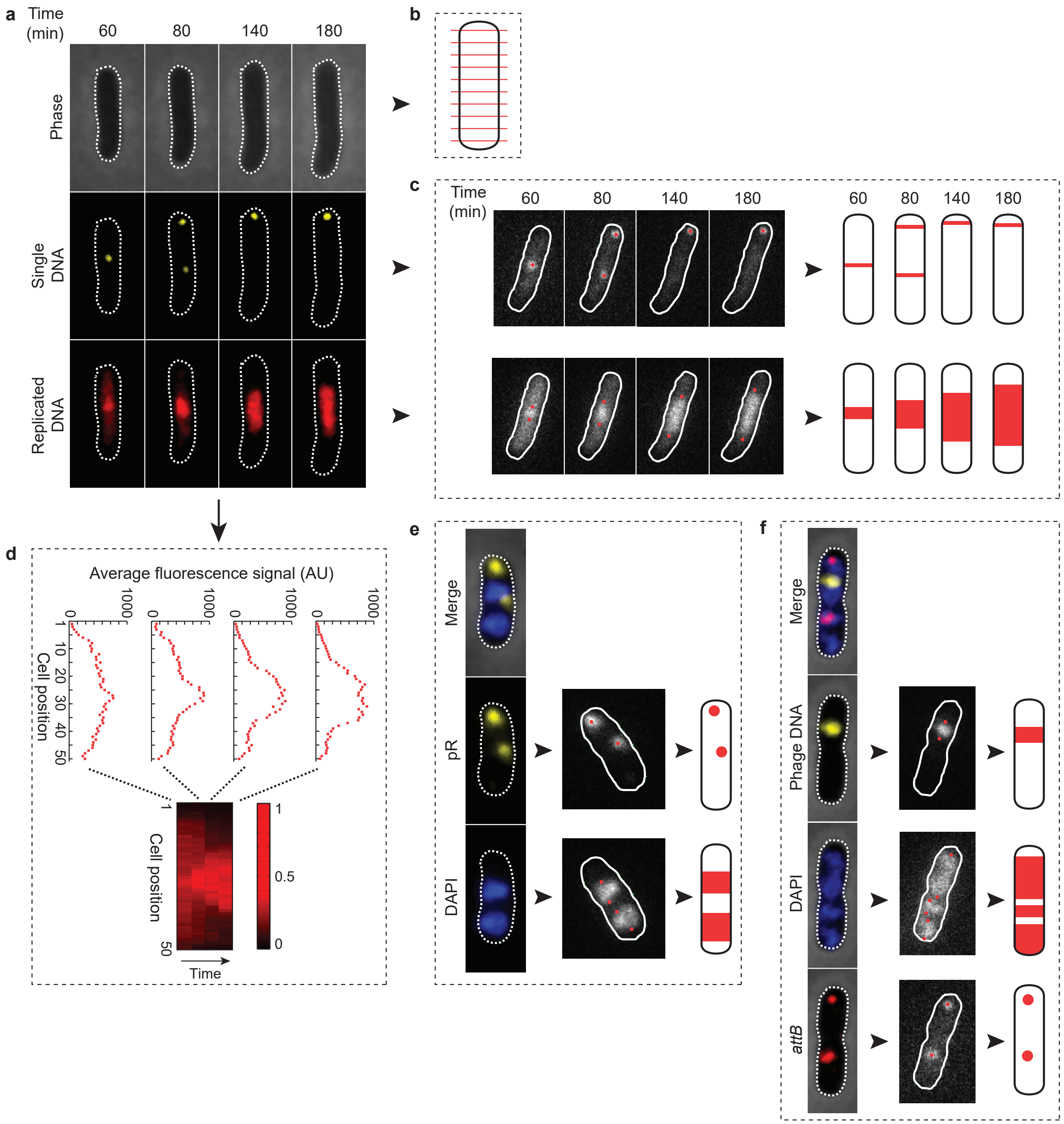

Supplementary Fig. 1. Overview of spatial analysis for live-cell infections, RNA FISH, and DNA FISH

a. Masks are drawn for cells using the phase-contrast channel. These masks are used to determine fluorescence locations in other channels. Example images from live-cell movies are shown.

b and **c.** For analysis, the cell mask is divided into equally thick slices (**b**), each representing a cell position for later analysis, along its major axis (50 slices). (**c**) The average fluorescence in

each slice is calculated. The data can then be converted into a heat map. The data are normalized either to the population maximum or within each cell to its own peak fluorescence, depending on the purpose of the figure.

(**d**) In all experiments, the location of fluorescence is determined using MicrobeTracker. In live-cell movies and lysogenic induction, the single DNA (SeqA) (not included in the induction experiment), DnaB, and capsid (gpD) foci are manually marked as spots in the program. These spots are then converted into the cell positions. For replicated DNA (TetR), the apparent boundaries of the cluster are manually marked in the MicrobeTracker, and then converted into the cell position of the cluster. Both the relative and absolute sizes can be calculated from this method.

(**e**) In RNA FISH experiments, DAPI locations are designated like TetR clusters in live-cell movies. For the transcripts, an additional step is performed for further analysis, where foci are manually marked and then converted into fixed-size circles within the boundaries of the cell mask. These areas are used to calculate the fluorescence in a 2D area.

(**f**) In DNA FISH experiments, both DAPI and phage DNA locations are determined like TetR clusters in live-cell movies. For the *attB* locations, the foci are treated like transcription clusters in RNA FISH experiments.

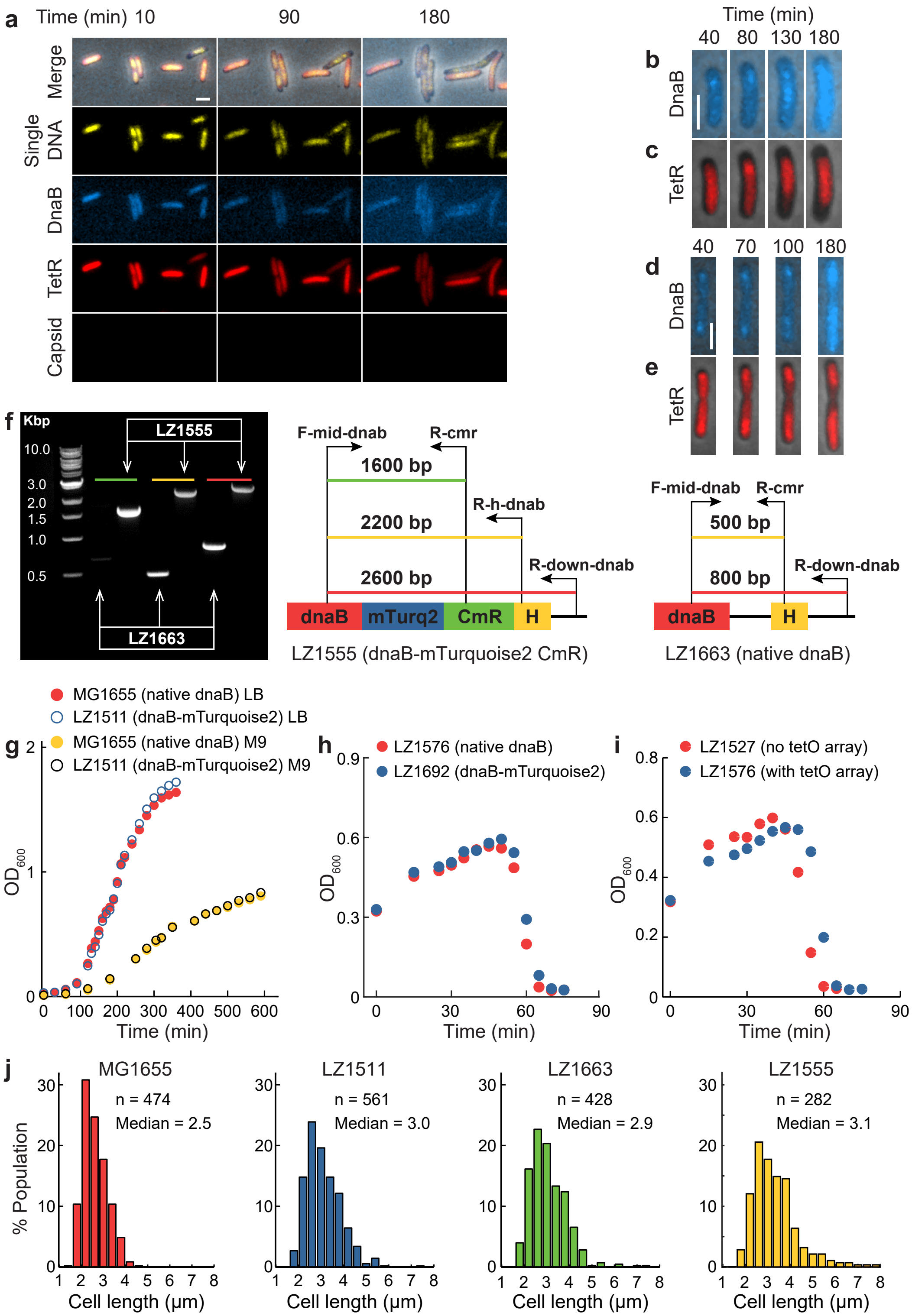

Supplementary Fig. 2. Fluorescent reporters are inserted correctly, do not negatively affect cell or phage growth, and do not aggregate in the absence of phage

a. Host cells for live-cell infection movies were imaged without phage infection. Contrast is fixed for each time point for each separate channel (see Supplementary Discussion). Representative cells chosen from three independent infection experiments. All scale bars in this figure are 2 μm.

b and **d.** Fixed-contrast images of the DnaB channel corresponding to Fig. 1e (**b**) and Fig. 1g (**d**). Supplemental movies also use fixed-contrast images for all channels and time points. Representative cells chosen from three independent infection experiments.

c and **e**. Fixed-contrast images of the Replicated DNA channel corresponding to Fig. 1e (**c**) and Fig. 1g (**e**). Representative cells chosen from three independent infection experiments.

f. PCR results for confirming insertion of the DnaB-mTurquoise2 reporter. A single forward primer within the *dnaB* gene was paired with three reverse primers in the antibiotic cassette (Cm^R, pair1 in green), homology region used for recombination (H, pair2 in yellow), or a location downstream of the construct which was not a part of any plasmids used for cloning (pair3 in red). The gel shows the results of PCR with these primers on the DnaB-mTurquoise2 strain (LZ1555) and the strain with the native *dnaB* (LZ1663).

g. Strains with the DnaB-mTurquoise2 construct grow the same as the parental strain. A growth curve of MG1655 and MG1655 with DnaB-mTurquoise2 (LZ1511) are shown for cells grown in LB or M9 media. The doubling time for MG1655 was calculated to be 26 min in LB and 82 min in M9. The doubling time for LZ1511 was calculated to be 26 min in LB and 86 min in M9.

h and **i.** Lysogen induction curves with DnaB-mTurquoise2 and *tetO* array constructs. (**h**) Isogenic reporter lysogens with (LZ1692) or without (LZ1576) the DnaB-mTurquoise2 construct were induced. The titer of the lysate for lysogens with DnaB-mTurquoise2 was 9.4E+9 pfu/ml. The titer of the lysate for lysogens without DnaB-mTurquoise2 was 9.2E+9 pfu/ml. (**i**) Isogenic reporter lysogens with (LZ1576) or without (LZ1527) the *tetO* array were induced. The titer of the lysate for lysogens with the *tetO* array was 9.2E+9 pfu/ml. The titer of the lysate without the *tetO* array was 1.5E+10 pfu/ml.

j. Size distributions of cells with and without the DnaB-mTurquoise2 construct. Cell sizes were determined by measuring cell length using phase-contrast images. MG1655 and MG1655 with DnaB-mTurquoise2 (LZ1511) are isogenic pairs to be compared. MG1655 SeqA-mKO2 *dam*$^-$ (LZ1663) and MG1655 SeqA-mKO2 *dam*$^-$ with DnaB-mTurquoise2 (LZ1555) are isogenic pairs to be compared.

Source data are provided as a source data file.

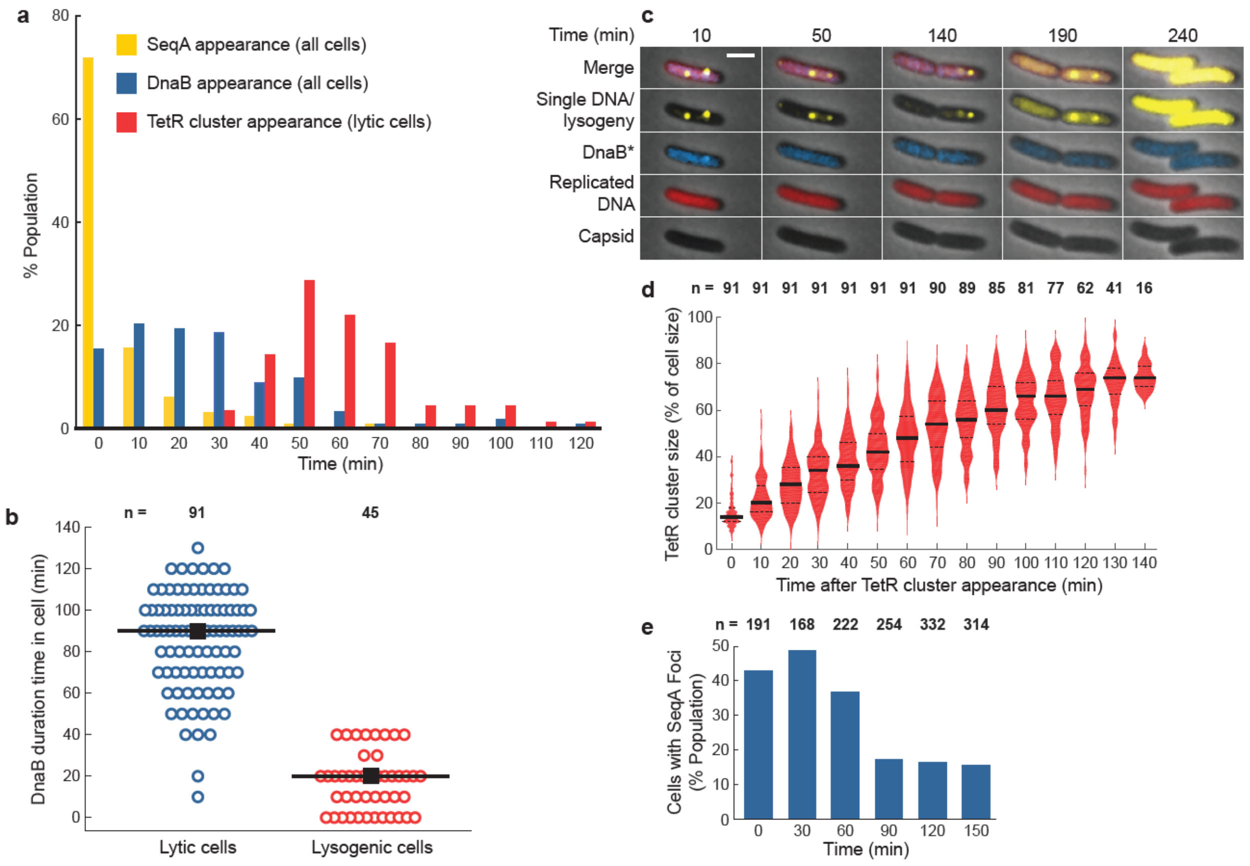

Supplementary Fig. 3. Fluorescent markers report phage development over time

a. Histograms of the first appearance of different reporters, yellow for SeqA, blue for DnaB, and red for TetR. For SeqA and DnaB, all cells are in the dataset, and for TetR, only lytic cells are included as lytic cells have extensive DNA replication. Time = 0 represents the first image taken during the movie, which occurs after processing the infected cells for imaging. Medians = 0 min (SeqA), 20 min (DnaB), and 60 min (TetR).

b. Lytic cells organize DnaB for longer time. The duration of DnaB foci is calculated by summing the time in which a DnaB focus was marked as present. The square with the line marks the median (90 min for lytic (blue), 20 min for lysogenic (red)). All scale bars in this figure are 2 μm.

c. Representative lysogenic cell grows and divides without lytic development. Representative cells chosen from three independent infection experiments. Organization of DnaB and TetR is less clear in lysogenic cells due to less DNA replication. The single DNA channel shows that phage DNA is in the cell, and the same channel is used for the lysogenic reporter. Due to the difference in timing, both the single DNAs' early behaviors and later lysogenic development can be observed despite sharing the same fluorescent protein (* indicates that contrast is adjusted for each time point shown for clarity).

d. Phage DNA fills the cell over time. The sizes of TetR clusters for lytic cells at different time points are represented as violin plots. Time = 0 when the TetR cluster is first observed, which varies per cell. The solid line represents the median and the dashed lines represent the interquartile range for violin plots.

e. SeqA foci from phage DNAs dissipate in dividing reporter cells. After infecting reporter cells (LZ1557) with methylated phages (λLZ1576) the number of cells with SeqA foci was tracked over time. SeqA decreases in the population as cells divide over time because Dam is not active.

Source data are provided as a source data file.

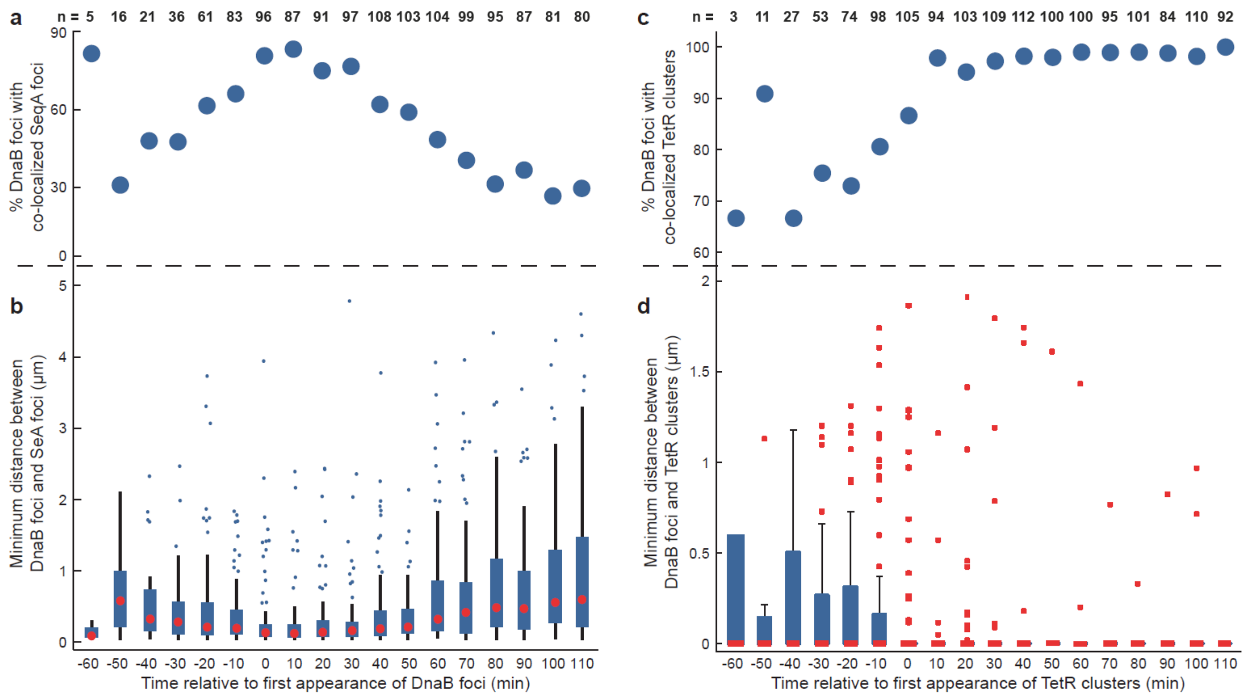

Supplementary Fig. 4. Phage DNA organizes DnaB before and during DNA replication

a and **b.** Single phage DNAs organize DnaB localization. (**a**) The percentage of co-localized SeqA and DnaB foci at each time point. Co-localization is defined as when the minimum calculated distance between a DnaB and SeqA focus is below 250 nm, an approximation of our resolution limit. Time is relative to each cell's first appearance of a DnaB focus (time = 0), so data from negative time points refer to the distance of SeqA at (time = t) from DnaB at (time = 0) as a means to measure how the position of SeqA changes before sequestering DnaB. (**b**) Boxplots of the distance data. The median is indicated by the dot at the center of the box, the box bounds the interquartile range of the data, the whiskers span the range of the data excluding the outliers, and the outliers are indicated as individual points.

c and **d.** Organization of resources and DNA replication persists over time. (**c**) The percentage of co-localized DnaB foci and TetR clusters at each time point. Co-localization is defined as when the minimum calculated distance between a DnaB focus and the outside edge of a TetR cluster is below 250 nm, an approximation of our resolution limit, or when a DnaB focus is within a TetR cluster. Time is relative to each cell's first appearance of a TetR cluster (time = 0), so data from negative time points refer to the distance of DnaB at (time = t) from a TetR cluster at (time = 0) as a means to measure how the position DnaB relates to where the replicated phage DNA cluster forms. (**d**) Boxplots of the distance data. The median is indicated by the dot at the center of the box, the box bounds the interquartile range of the data, the whiskers span the range of the data excluding the outliers, and the outliers are indicated as individual points. The median distance between DnaB and TetR is zero for all time points. Zero is the specific case when DnaB and TetR are designated as overlapping.

Source data are provided as a source data file.

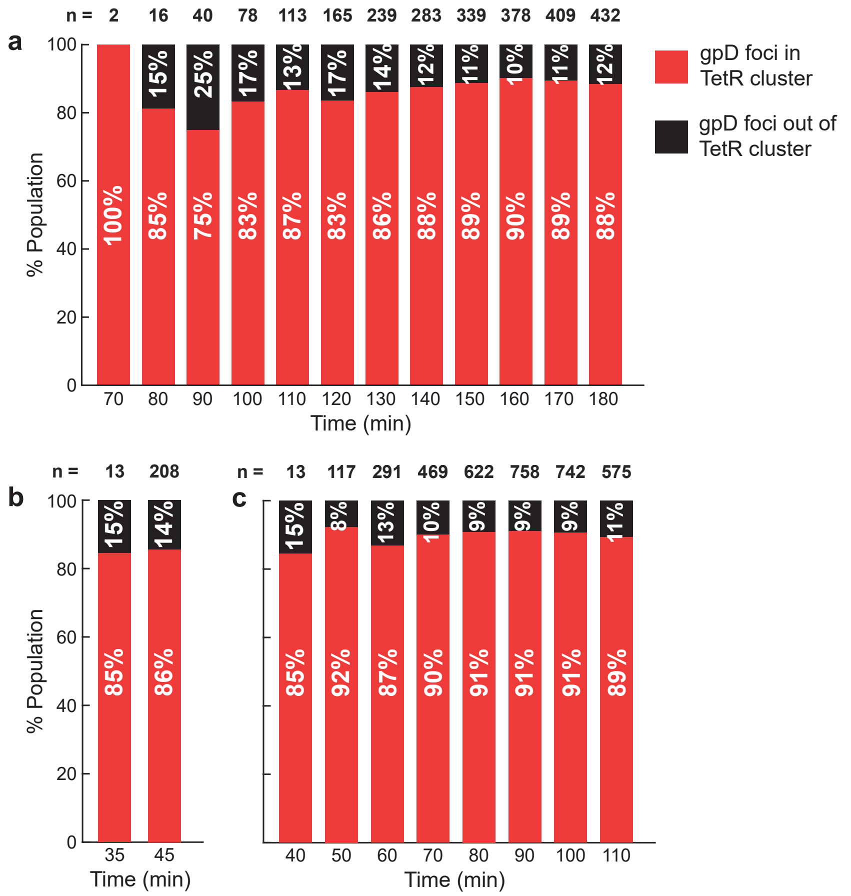

Supplementary Fig. 5. Phage virions assembled in phage DNA clusters

a - c. Assembled virions localize with phage DNA. The percentage of gpD foci located inside and outside the designated boundaries of TetR clusters is shown at time points with gpD foci for live-cell movies (**a**, from Fig. 1), lysogen induction (**b**, from Fig. 2), and live-cell movies with the *attB* reporter (**c**, from Fig. 3).

Source data are provided as a source data file.

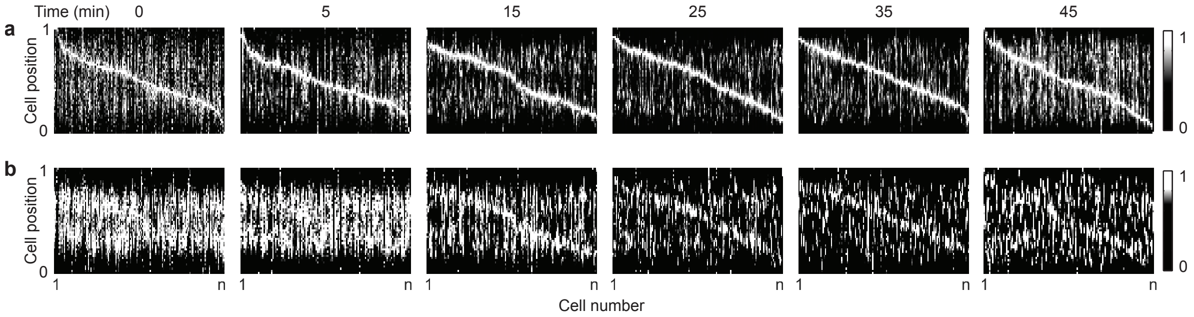

Supplementary Fig. 6. Organization of multiple heterogeneous subcellular areas during phage development

a and **b.** Heat maps of DnaB signal are arranged according to the location of its peak intensity for each time point (**a**). Fluorescence for each cell is normalized to its own peak intensity for (**a-b**). (**b**) Heat maps of TetR signal are arranged in the same order as in (**f**) to compare the locations of DnaB and TetR. Only after induction has progressed phage DNA replicates and becomes organized. n = 155, 118, 137, 155, 148, and 121 for the increasing time points.

Source data are provided as a source data file.

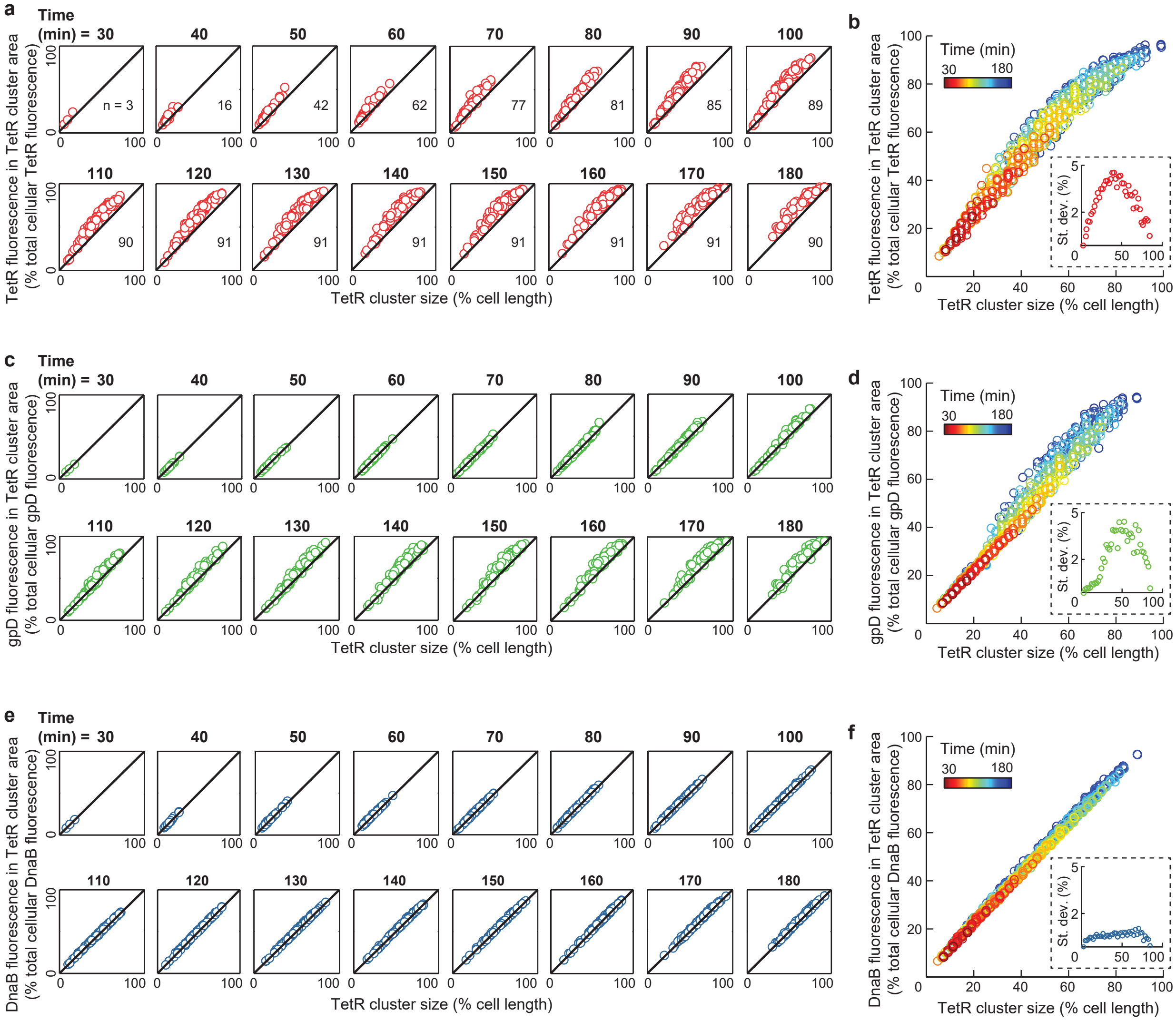

Supplementary Fig. 7. Subcellular DNA replication and phage assembly processes are heterogeneous (LZ1557 and phage λLZ1576)

a. For each time point, the size of the phage DNA cluster is plotted against the percentage of the total cellular replicated DNA-reporter fluorescence residing in the cluster. The black line represents a slope = 1.

b. The data from (**a**) are combined and plotted with different colors on a single plot, showing the change in fluorescence distribution over time. Data are slightly offset along the x-axis for visibility. (**b**, inset) The standard deviation of the y-values is plotted for each x-value in (**b**) to show the heterogeneity of the phage process reported by the fluorescence signal.

c and **d.** Same as (**a** and **b**), except the capsid fluorescence is plotted against the cluster size to show how capsid fluorescence prefers to localize. Data are slightly offset along the x-axis for visibility.

e and **f.** same as (**a** and **b**), except plotting DnaB fluorescence. Data are slightly offset along the x-axis for visibility. This serves as a diffuse control because DnaB fluorescence is relatively diffuse due to the DnaB foci being not particularly brighter than the background fluorescence of the cell.

Source data are provided as a source data file.

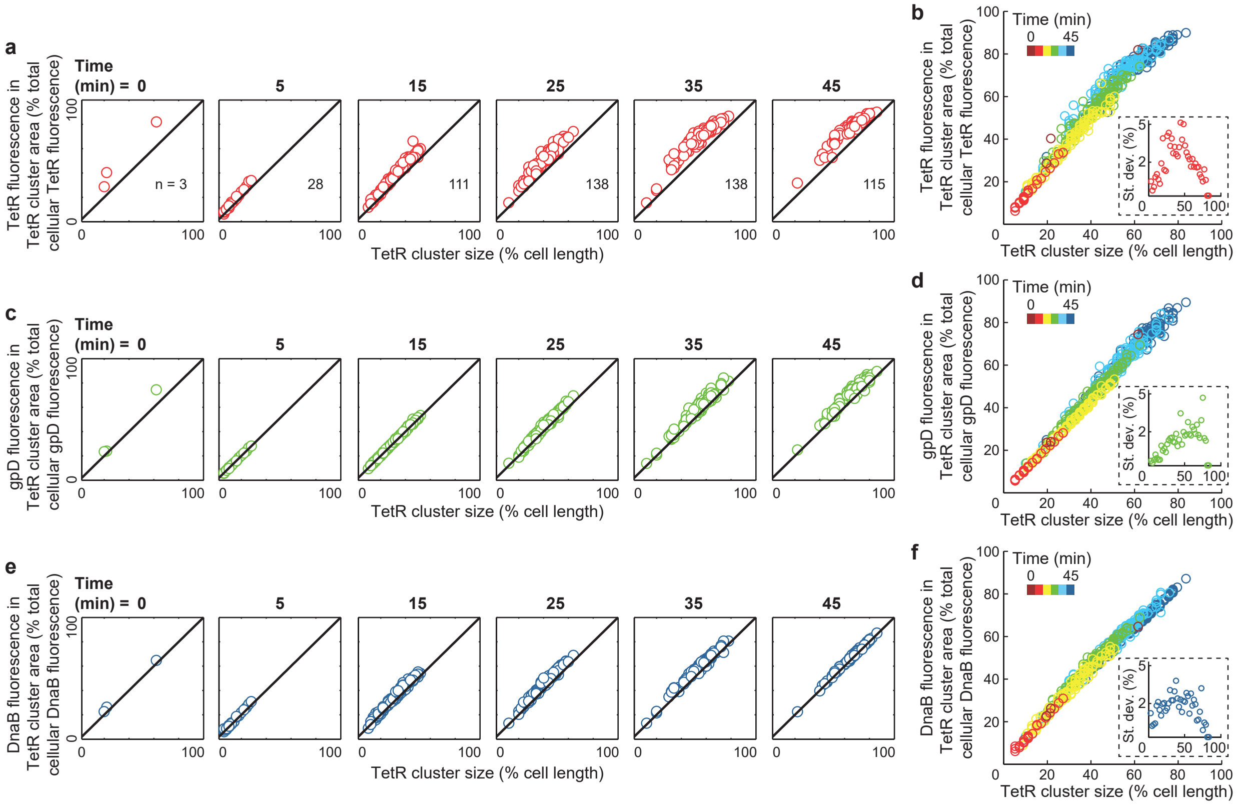

Supplementary Fig. 8. Subcellular DNA replication and phage assembly processes are heterogeneous (LZ1596 induction)

a - f. These panels are similar to Supplementary Fig. 7, except it is using the data from the induction experiment. Notably, DnaB does behave slightly differently here than in the live-cell movies. DnaB's increase in organization can be attributed to the different experimental conditions. Inductions were grown in LB, increasing the amount of DnaB in the cell, allowing clusters to have stronger signal over background, and cells were not imaged over time, so fluorescence is stronger in general without photobleaching.

Source data are provided as a source data file.

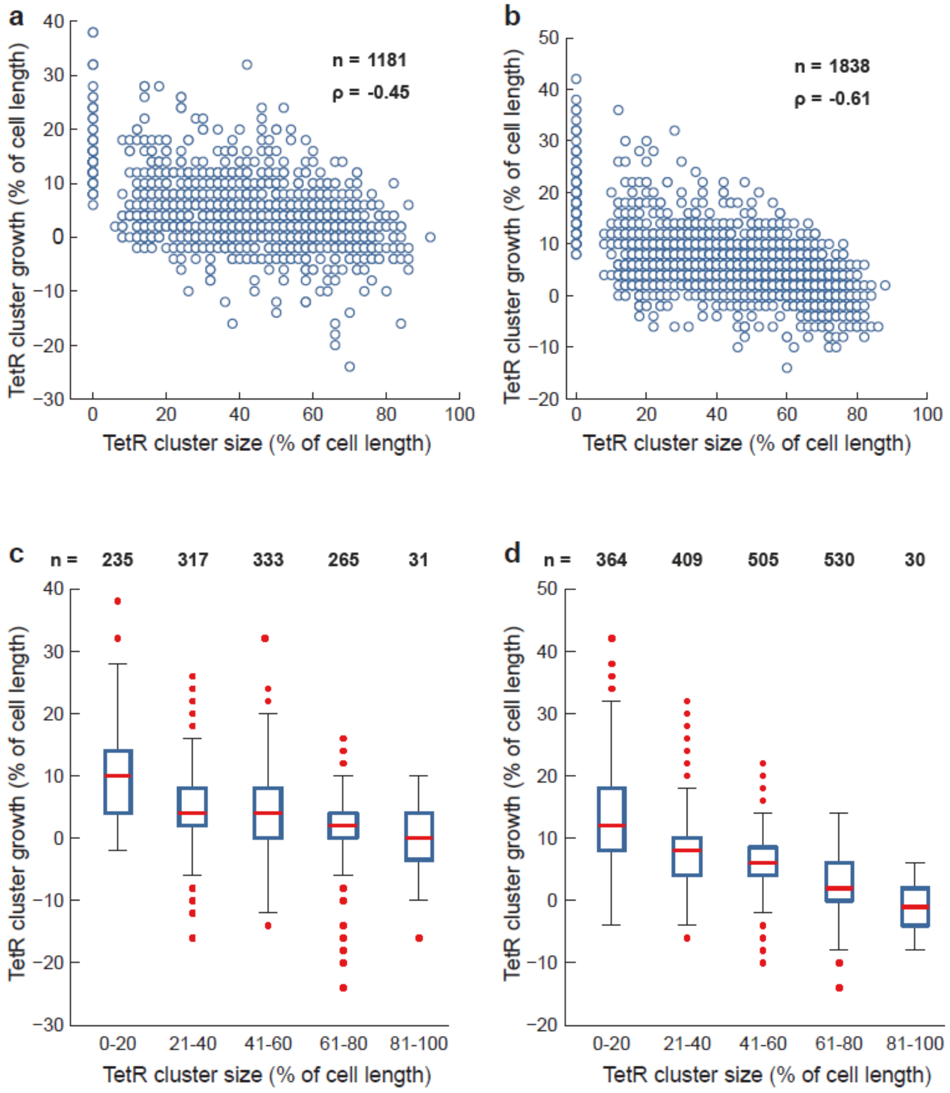

Supplementary Fig. 9. Expanse of replicating phage DNA is limited

a - d. Phage DNA expansion decreases as it fills the cell. The growth of the phage DNA cluster is calculated as the difference between the calculated size of the cluster at time t and t+1. (**a-b**) Each growth is plotted against the size of the cluster at time t, to compare how the growth of phage DNA clusters depends on the amount of phage DNA-free space in the cell. Negative growth means that the cluster shrinks between the time points. Growth negatively correlates with cell occupancy (Pearson's ρ in plots, p-values < 0.001 for (**a**) and (**b**)). Boxplots in (**c** and **d**) are shown for each bin of DNA size. (**a** and **c**) Data from the live-cell infection movies using LZ1557 and phage λLZ1576, Fig. 1. (**b** and **d**) data from the infection movies using LZ1643 and phage λLZ1629, Fig. 3. For the boxplots, the median is indicated by the line at the center of the box, the box bounds the interquartile range of the data, the whiskers span the range of the data excluding the outliers, and the outliers are indicated as individual points.

Source data are provided as a source data file.

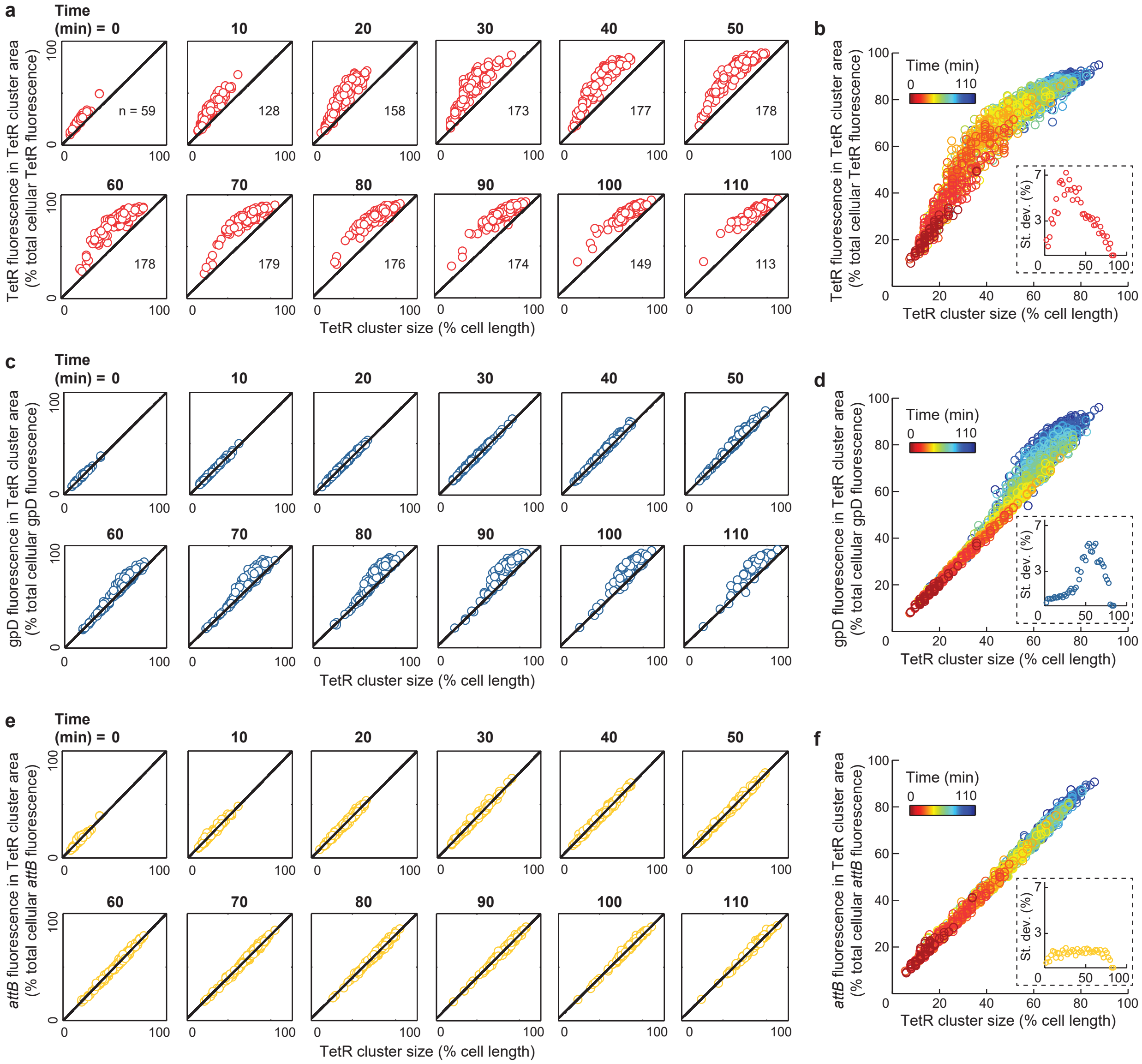

Supplementary Fig. 10. Subcellular DNA replication and phage assembly processes are heterogeneous (LZ1643 and phage λLZ1629)

a - f. Same as in Supplementary Fig. 7, except these data are from the live-cell movies using the *attB* strain as in Fig. 3. In these movies, the *attB* reporter is relatively diffuse because the foci are not much brighter than the background cell fluorescence and behaves similarly to a diffuse control.

Source data are provided as a source data file.

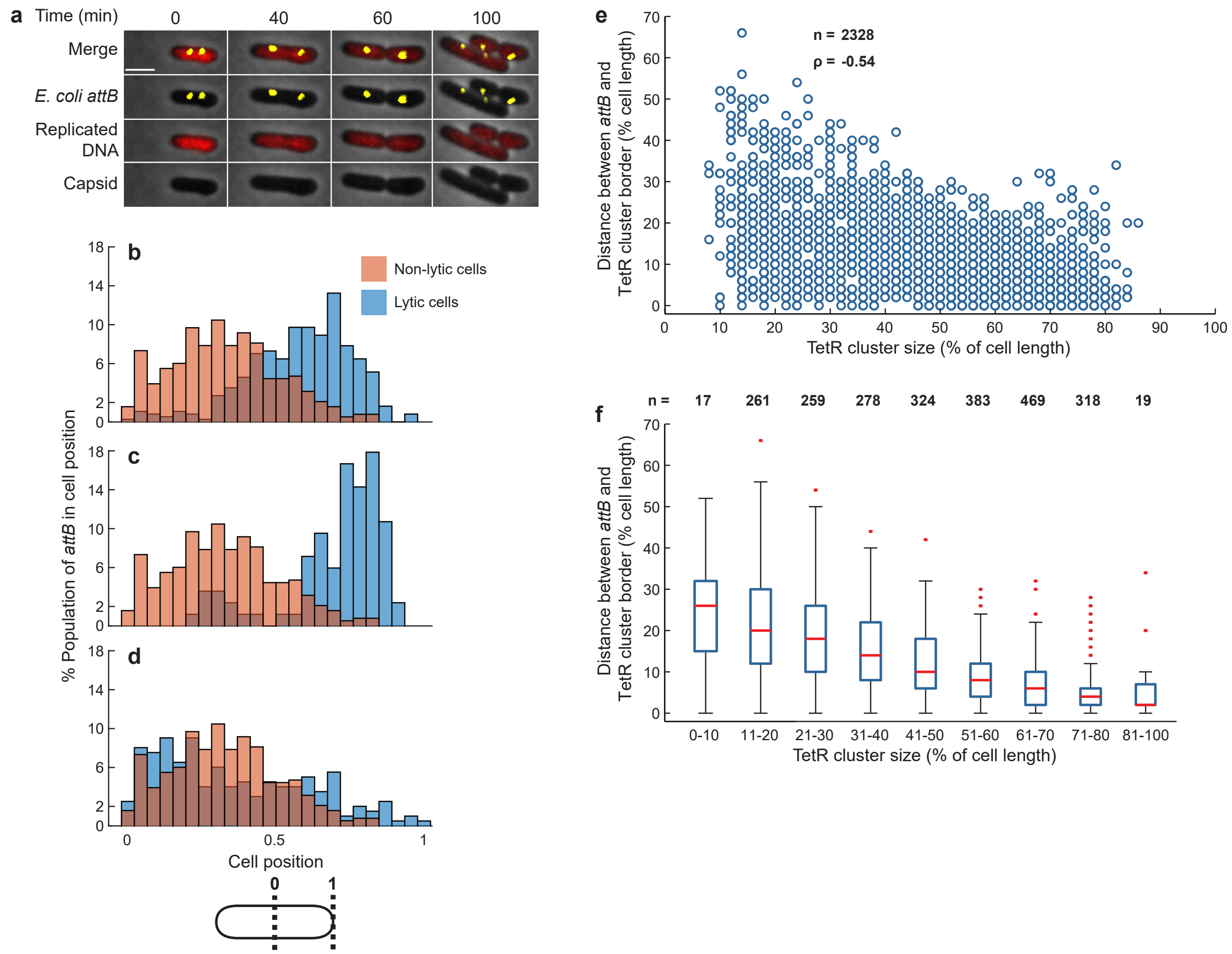

Supplementary Fig. 11. Phage DNA is physically separate from E. coli DNA which affects the organization of both species

a. *E. coli attB* migrates to the quarter-cell position before cell division in the absence of lytic development. Example images are shown of cells which divide. No reorganization of the replicated DNA reporter occurs without lytic development. Representative cells chosen from four independent infection experiments. All scale bars in this figure are 2 µm.

b - d. Lytic development force changes in *attB* localization. The lytic cells' data from Fig. 3h is divided into the lytic subcategories of push (**b**, 370 data points), spread (**c**, 84 data points), and squeeze (**d**, 199 data points), and compared to the non-lytic data to show how specific types of lytic development change *attB* organization. Push and spread development biases *attB* to the poles, whereas squeeze development biases *attB* toward the mid-cell. As squeezing requires multiple phage DNA clusters, the positions of those clusters will determine where the *attB* marker is squeezed within. The cell below shows how location is represented.

e and **f.** Replicating phage DNA avoids *E. coli attB*. The absolute minimum distance between the *attB* location and the designated boundary of the phage DNA clusters is calculated and

plotted against the size of the clusters. (**e**) The size of the phage DNA cluster negatively correlates with the distance between *attB* and phage DNA (Pearson's ρ in plot, p-value < 0.001). (**f**) Boxplots are shown for each bin of DNA cluster size. The median is indicated by the line at the center of the box, the box bounds the interquartile range of the data, the whiskers span the range of the data excluding the outliers, and the outliers are indicated as individual points. The behavior shows that the cluster edge moves closer to *attB* as DNA grows, and the distance remains small as DNA continues to grow. These data include all of the different interactions of phage DNA and *attB* instead of just the push subclass in Fig. 3i-j.

Source data are provided as a source data file.

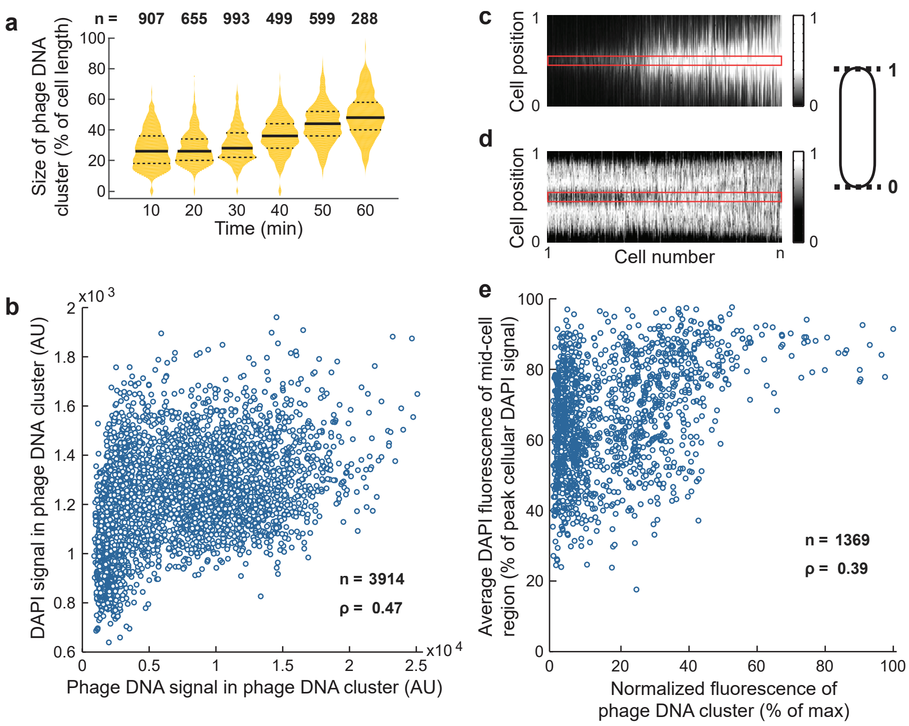

Supplementary Fig. 12. Phage DNA replicates over time in subcellular areas

a. Phage DNA occupies more of the cell over time with more DNA replication. Violin plots of phage DNA size relative to cell length show how phage DNA fills the cell in DNA FISH experiments. In the violin plots, the solid line represents the median, and the dashed lines mark the bounds of the interquartile range of the data.

b. The DAPI fluorescence within the designated phage DNA cluster for each cell is plotted against the phage DNA fluorescence within that cluster. Increasing phage DNA signals positively correlate with increased DAPI signals (Pearson's ρ in plot, p-value < 0.001).

c - e. Phage DNA is only detectable via DAPI staining at high phage DNA levels. (**c**) Heat maps of cells, with a single designated DNA cluster crossing the mid-cell, are arranged by increasing phage DNA signal. The fluorescence of these cells is normalized to the population maximum. (**d**) Heat maps of the DAPI signals of the cells, in the same order, as in (**c**) are stacked to show how the DAPI signals change compared to the phage DNA signal. The fluorescence of these cells is each normalized to the population maximum. The cell to the right shows how location is represented for (**c-e**). The red box in (**c** and **d**) outlines the middle 10% of each cell to show where the signals are being calculated and plotted in (**e**). The DAPI fluorescence in the mid-cell are plotted against the phage DNA signal in the mid-cell, showing a positive correlation (Pearson's ρ in plot, p-value < 0.001). n = 1369 cells for (c and d).

Source data are provided as a source data file.

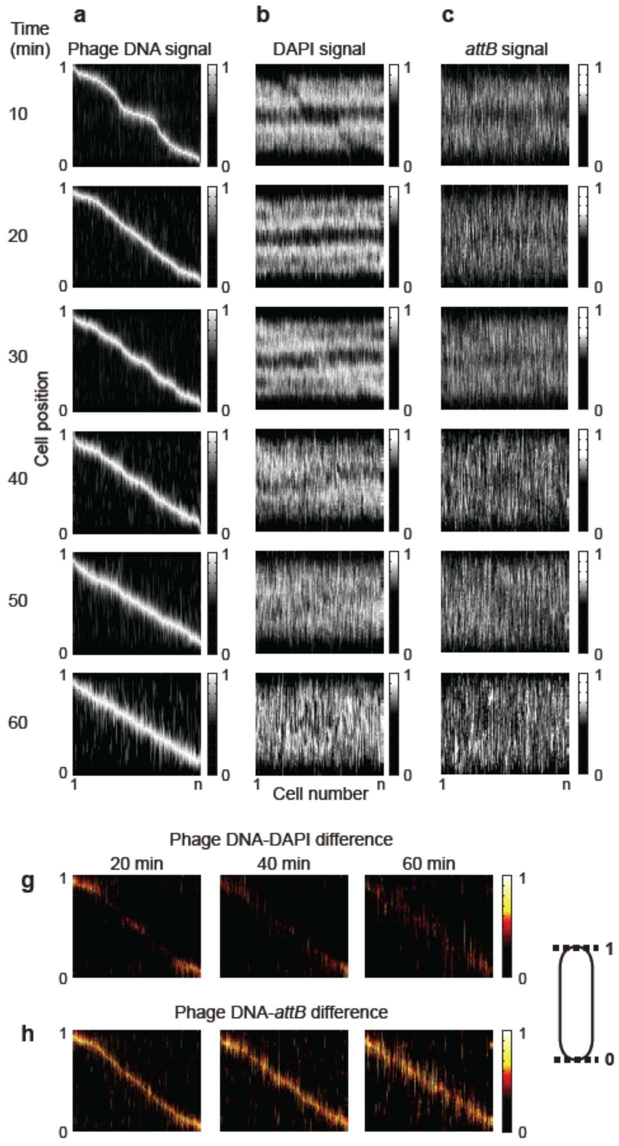

Supplementary Fig. 13. Phage DNA replicates preferentially in E. coli DNA-free locations and reorganizes bacterial DNA

a - c. Related to Fig. 4j-k. Heat maps of phage DNA (**a**), DAPI (**b**), and *attB* (**c**) signal at given time points are arranged by location of peak intensity. The fluorescence of each cell is normalized to its own peak brightness for (**a-f**) For each respective time point, the difference map in Fig. 4j is equal to (**a**)-(**b**), and the difference map in Fig. 4k is equal to (**a**)-(**c**). Sample size n in (**a-c**) is the same as that in Fig. 4b at the respective time points.

d - f. *attB* co-localizes with E. coli DNA signal. (**d**) Heat maps of the attB signal of the cells from the DNA FISH experiments in Fig. 4 are arranged by location of peak intensity at given time points. (**e**) Heat maps of DAPI signal are arranged in the same order as in (**d**) to compare DAPI signals with *attB* signals. (**f**) Difference maps, calculated as (**d**)-(**e**), are shown to compare DAPI signals to the location of *attB* signals. Negative values are set to zero.

g and **h.** Difference maps between phage DNA-DAPI (**g**) and phage DNA-*attB* (**h**), as in Fig. 4j-k, at the time points not shown in Fig. 4j-k. The cell to the right shows how location is represented for (**a-h**).

Source data are provided as a source data file.

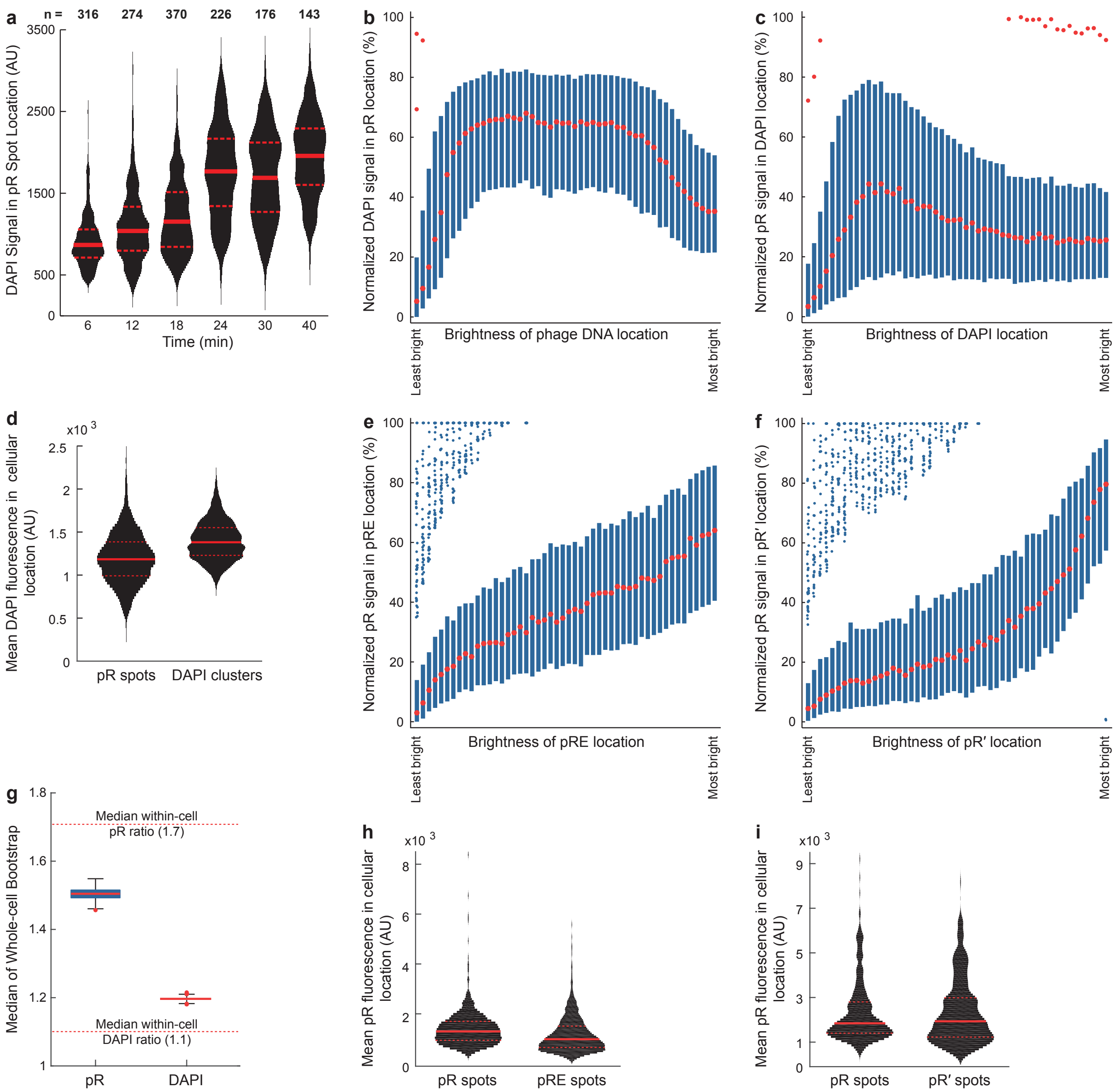

Supplementary Fig. 14. Phage gene expression co-localizes with phage DNA and phage decisions are enacted in subcellular locations

a. Locations of pR spots have increased DAPI staining over time. The DAPI signal in designated pR spots for cells at different time points after infection. Increased signals suggest DNA is more stained at the locations. In the violin plots, the solid line represents the median, and the dashed lines mark the bounds of the interquartile range of the data.

b and **c.** Strong pR signals anti-correlate with DAPI signals. (**c**) The normalized pR signals (data from Fig. 4l) for each cell are sorted by intensity. The normalized DAPI signals (data from Fig. 4m) for each cell were sorted in the same order as the sorted pR signals. The DAPI data

corresponding to each pR location of relative brightness is plotted in boxplots. The median is indicated by the dot at the center of the box, the box bounds the interquartile range of the data, and the outliers are indicated as individual points. For these boxplots, the range of the data (which would be whiskers running through the box) is removed for plot-readability. Practically all of the ranges run from 0–100%, making the actual visualization of the ranges unnecessary. For the boxplots with outliers, the range would run to the outliers. (**c**) Same as (**b**), except initially sorting by DAPI signals. n = 2035 for (**b-c**).

d. Phage mRNA locates to areas without *E. coli* DNA. Violin plots of the fluorescence of DAPI signals inside designated pR spot areas (left) and inside designated DAPI clusters (right). In the violin plots, the solid line represents the median, and the dashed lines mark the bounds of the interquartile range of the data. DAPI within pR is significantly lower than in DAPI clusters (Wilcoxon rank sum = 3150422, p-value < 0.001). n = 2035 cells.

e and **f.** Normalized pRE signals for each cell are sorted by intensity (**e**) Normalized pR signals for each cell were sorted in the same order as the pRE signals. pR intensity at each pRE location of relative brightness is plotted in boxplots. The median is indicated by the dot at the center of the box, the box bounds the interquartile range of the data, and the outliers are indicated as individual points. For these boxplots, only the interquartile range, median, and outliers are included, for readability. n = 645 cells with both pR and pRE spots. (**f**) Similar to (**e**) except initially sorting by pR'. n = 439 cells with both pR and pR' spots.

g. Red dashed lines represent the median ratio of averaged signals between cell halves, a measure of within-cell variability, 1.7 for pR and 1.1 for DAPI (n = 2035). The boxplots represent the distribution of medians resulting from 1000 bootstrap runs that randomly paired whole-cell averaged signals (n = 2035), which measures between-cell variability. The median is indicated by the line at the center of the box, the box bounds the interquartile range of the data, the whiskers span the range of the data excluding outliers, and the outliers are indicated as individual points.

h. Levels of pR mRNA are lower where pRE is present. Violin plots of the fluorescence of pR signals inside designated pR spot areas (left) and inside designated pRE spot areas (right). In the violin plots, the solid line represents the median, and the dashed lines mark the bounds of the interquartile range of the data. Median pR within pRE is significantly lower than in pR clusters (Wilcoxon rank sum = 455253, p-value = 2.3e-16 two-tailed). n = 645 cells with both pR and pRE spots.

i. The pPR' transcripts localize to pR transcript locations. Violin plots of the fluorescence of pR signals inside designated pR spot areas (left) and inside designated pR' spot areas (right). In the violin plots, the solid line represents the median, and the dashed lines mark the bounds of the interquartile range of the data. Median pR within pR' is not significantly different from median pR within pR spots (Wilcoxon rank sum = 194182, p-value = 0.74 two-tailed). n = 439 cells with both pR and pR' spots.

Source data are provided as a source data file.

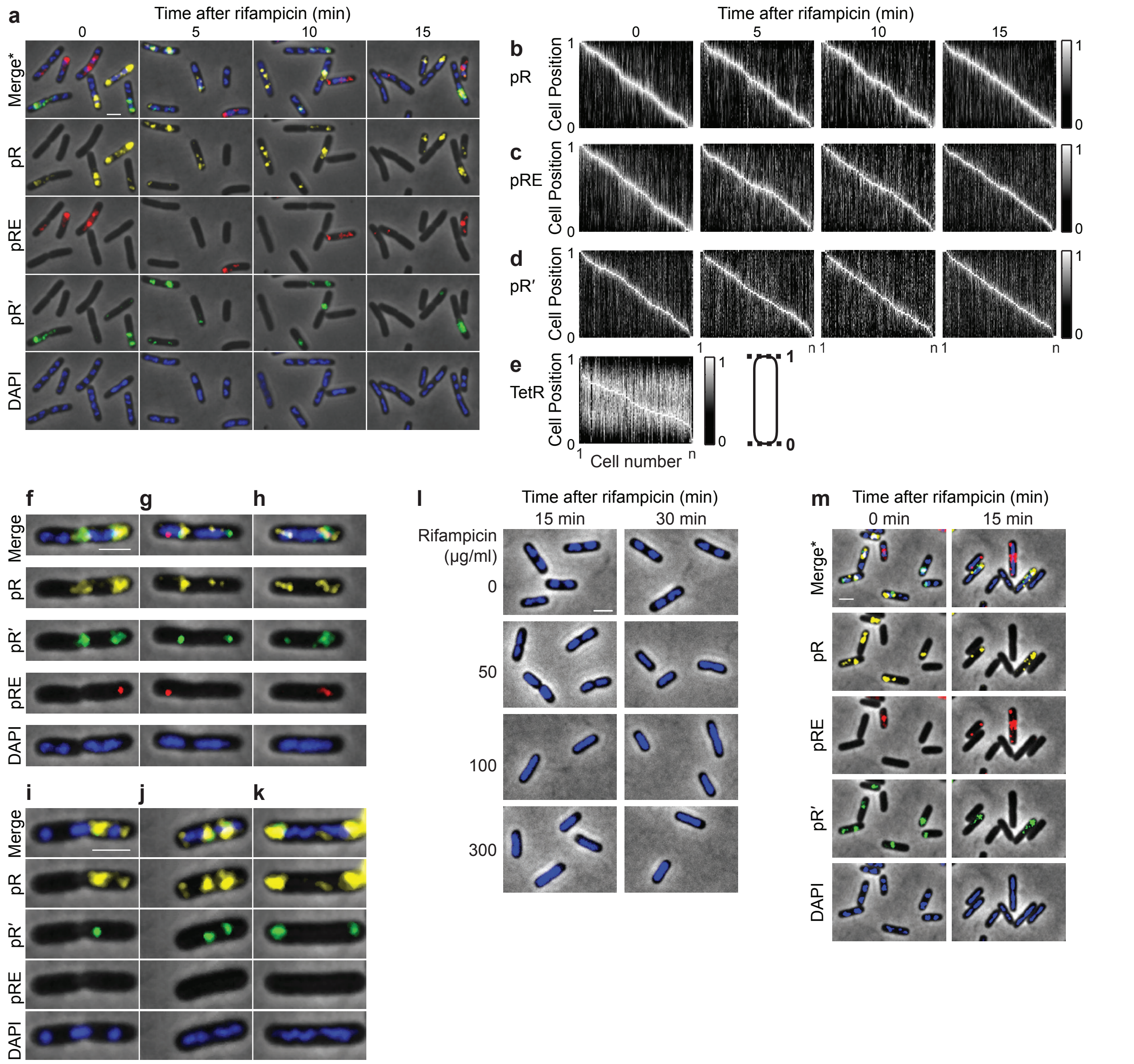

Supplementary Fig. 15. Phage mRNAs retain localization after completing transcription

a. Phage mRNAs remain localized after transcription initiation is inhibited. Representative images of cells show clusters of phage transcripts (pR, pRE and pR') and DAPI signal remaining organized after treatment with rifampicin. Representative cells chosen from four independent infection experiments. Rifampicin blocks further transcription initiation, so active transcripts continue and terminate over time. Remaining signal over time represents completed transcripts. *This figure is not a time lapse, so variable contrast was used for each time point as FISH intensity decreases over time as mRNAs degrade. All scale bars in this figure are 2 µm.

b - d. Heat maps of each transcript show the maintenance of mRNA localization over time in the absence of new transcription. FISH signals from each transcript remain similarly localized after

treatment with rifampicin, indicating that after transcription terminates, phage mRNAs remain in clusters. n = 489, 345, 285, and 541 cells for time points 0, 5, 10, and 15 min respectively.

e. Heat map of TetR signal in non-induced lysogens represents how non-localized signals appear in a population. These data are related to Supplementary Fig. 6b at 0 min. TetR freely diffuses when there is little phage DNA, so the heat maps appear more uniform compared to the FISH data, where the localization of phage mRNAs over time result in heat maps with contrasting dark and light regimes. This figure is meant to compare how localized vs. non-localized signals appear within a population. The same contrast was used for each heat map in (**b-e**). The cell to the right shows how location is represented for (**b-e**). n = 155 cells.

f - k. Separation of pRE and pR' in different phactories in same cell is maintained without further transcription. (**f-h**) Representative images of cells after 15 min of rifampicin show separated pRE and pR' clusters in single cells, similar to Fig. 5f-g (occurs in 21/489 at 0 min, 13/345 at 5 min, 14/285 at 10 min, and 21/541 cells at 15 min after rifampicin addition). Representative cells chosen from four independent infection experiments. (**i-k**) Representative images of cells after 15 min of rifampicin treatment show fewer separated pR' clusters than pR clusters in single cells, similar to Fig. 5d-e (occurs in 32/489 at 0 min, 22/345 at 5 min, 19/285 at 10 min, and 27/541 cells at 15 min after rifampicin addition). Separate decisions occur in continuously separated intracellular phactories.

l. Increasing dosages or exposure time to rifampicin induces nucleoid expansion. Cells were grown and treated with the indicated amount of rifampicin and then images were taken after the indicated amount of time for each group. With 15 µg/ml of rifampicin, 63/151 cells retained compacted nucleoids at 15 min, and 20/123 cells retained compacted nucleoids at 30 min. With 100 µg/ml of rifampicin, 39/161 cells retained compacted nucleoids at 15 min, and 11/121 cells retained compacted nucleoids at 30 min. With 300 µg/ml of rifampicin, 17/192 cells retained compacted nucleoids at 15 min, and 1/79 cells retained compacted nucleoids at 30 min. The compaction of nucleoid is guided by the eye. Representative cells chosen from three independent infection experiments.

m. Phage mRNAs remain localized after transcription initiation is inhibited with increased rifampicin. Representative images of cells show clusters of phage transcripts (pR, pRE, and pR') and DAPI signal remaining organized after treatment with 300 µg/ml of rifampicin. *This figure is not a time lapse, so variable contrast was used for each time point as FISH intensity decreases over time as mRNAs degrade. Representative cells chosen from three independent infection experiments.

Source data are provided as a source data file.

Strain #	Bacterial Strains	Comments	Source
-	MG1655	Wild type *E. coli*	Lab stock
LZ1383	MG1655 *seqA-mKO2 Cm^R-FRT*	Strain with SeqA fusion	This work
LZ1386	MG1655 *seqA-mKO2 Cm^R-FRT Δdam::Kan^R*	Strain with SeqA reporter, *dam* deletion donor	Sup. ref. [9]
LZ1510	MG1655 *dnaB-mTurquoise2 Cm^R-FRT*	Strain with DnaB reporter	This work
LZ1511	MG1655 *dnaB-mTurquoise2*	Strain with DnaB reporter, no Cm marker	This work
LZ1527	MG1655 (*λD-mNeongreen cI_{857}-mKO2 bor::Kan^R*) [pBR322 pLate**D*]	Lysogen without a 24x *tetO* array	This work
LZ1535	MG1655 seqA-mKO2	Strain with SeqA fusion, no Cm marker	This work
LZ1552	MG1655 *seqA-mKO2 dnaB-mTurquoise2 Cm^R-FRT*	Strain with SeqA fusion and DnaB reporter	This work
LZ1555	MG1655 *seqA-mKO2 dnaB-mTurquoise2 Cm^R-FRT Δdam::Kan^R*	Strain with full SeqA and DnaB reporters	This work
LZ1557	MG1655 *seqA-mKO2 dnaB-mTurquoise2 Cm^R-FRT Δdam::Kan^R* [pACYC177 pFtsKi *tetR-mCherry*]	Triple reporter strain (SeqA, DnaB, and TetR plasmid) for phage infection	This work
LZ1575	MG1655 (*λD-mNeongreen cI_{857} bor::Cm^R 24x tetO*) [pBR322 pLate**D*]	Lysogen, induced to produce λLZ1575	This work
LZ1576	MG1655 (*λD-mNeongreen cI_{857}-mKO2 bor::Cm^R 24x tetO*) [pBR322 pLate**D*]	Lysogen, induced to produce λLZ1576	This work
LZ1596	MG1655 *dnaB-mTurquoise2* (*λD-mNeongreen cI_{857} bor::Cm^R* 24x *tetO*) [pACYC177 pFtsKi *tetR-mCherry*]	Lysogen with prophage (λLZ1575), with DnaB and replicated phage DNA reporter	This work
LZ1629	MG1655 (*λD-mTurquoise2 cI_{857} bor::Cm^R 24x tetO*) [pACYC177 pLate**D*]	Lysogen, induced to produce λLZ1629	This work
LZ1643	MG1655 *attB-96x lacO Kan^R* [pACYC177 pFtsKi *tetR-mCherry lacI-eyfp*]	Reporter strain with *attB* marker, plasmid labels *tetO* and *lacO* sites	This work
LZ1663	MG1655 *seqA-mKO2 Δdam::Kan^R*	Strain with a native *dnaB* allele	This work
LZ1692	MG1655 *dnaB-mTurquoise2* (*λD-mNeongreen cI_{857}-mKO2 bor::Cm^R 24x tetO*) [pACYC177 pLate**D*]	Lysogen with a *dnaB-mTurquoise2* allele	This work
	**Phage Strains**		
λLZ613	*λcI_{857} bor::Kan^R*	Wild type phage, no fluorescence reporters	Lab stock
λLZ1269	*λD-mTurquoise2 cI_{857} bor::Kan^R*	Phage with gpD reporter	This work
λLZ1369	*λD-mNeongreen cI_{857} bor::Kan^R*	Phage with gpD reporter	Sup. ref. [9]
λLZ1527	*λD-mNeongreen cI_{857}-mKO2 bor::Kan^R*	Phage with gpD and CI reporters	This work
λLZ1575	*λD-mNeongreen cI_{857} bor::Cm^R 24x tetO*	Phage with gpD reporter, *tetO* array	This work
λLZ1576	*λD-mNeongreen cI_{857}-mKO2 bor::Cm^R 24x tetO*	Phage with gpD and CI reporters, *tetO* array	This work
λLZ1629	*λD-mTurquoise2 cI_{857} bor::Cm^R 24x tetO*	Phage with gpD reporter, *tetO* array	This work
	**Plasmids**		
	pACYC177 or pBR322 pLate**D*	pLate**D* expresses gpD protein under native phage control, improves phage stability	Lab Stock
	pACYC177 pFtsKi *tetR-mCherry*	Expresses TetR fusion to bind to *tetO*, weakly constitutive. Referred to as TetR-mCherry	Gift of D.J. Sherratt
	pACYC177 pFtsKi *tetR-mCherry lacI-eyfp*	Expresses TetR fusion to bind to *tetO* and LacI fusion to bind to *lacO*, weakly constitutive	Gift of D.J. Sherratt
	PCP20	Temperature sensitive plasmid to recombine flanking FRT sites to remove DNA inserts	Lab Stock
	pKD46	Red-recombination plasmid	Lab Stock
	pBR322 24x *tetO bor::Cm^R*	*tetO*-recombination plasmid	This work
	p*dnaB-mTurquoise2-Cm^R*-H	Contains linear DNA used for red-recombination to make DnaB reporter	This work
	p*attB* 96x *lacO Kan^R*	Contains linear DNA used for red-recombination to make *attB* reporter	This work

Supplementary Table 1. Bacteria, phages and plasmids used in this study

SRRz _1	5'-CGGTATTCGCTTAATTCAGC		*SRRz* _24	5'-GGGTGATCGGAGTAATCAGT
*SRRz*_2	5'-TGTACGCATTTGGTGATCCG		*SRRz* _25	5'-GTTTAGCGTGACAAGTTTGC
SRRz _3	5'-CAGTTTGGGTTGTGCTGTTG		*SRRz* _26	5'-CGCCTGTTGATTTGAGTTTT
SRRz _4	5'-AATTCAGGACAGACAGTGGC		*SRRz* _27	5'-GAGAAGTCTTTCAGGCCAAG
SRRz _5	5'-CCGCAGCGTAACTATTACTA		*SRRz* _28	5'-GCTCCTTAATCTGCTGCAAT
SRRz _6	5'-CTTTCACGAAGGTCATGTGT		*SRRz* _29	5'-TCAATCATAGGTAAAGCGCC
SRRz _7	5'-CAAAACGGCAGGAGGTTGTT		*SRRz* _30	5'-GATTGCCTGACGGATATCAC
SRRz _8	5'-TTTGTTCGTGACCGATATGC		*SRRz* _31	5'-GCAGTGAAGCCCAGATATTG
SRRz _9	5'-CAGGCTACTGTGTTTAGTAA		*SRRz* _32	5'-ATCAGGCTGTCAGCCTTATG
SRRz _10	5'-GGTCGATTACTGATAGAACA		*SRRz* _33	5'-GCCCGCTTCTTTGAATTTTG
SRRz _11	5'-TGGCATCTTCATGTCTTACC		*SRRz* _34	5'-TACATCAATCTCTCTGACCG
SRRz _12	5'-CGGCCAACAGGTCATGTTTT		*SRRz* _35	5'-AGATAATCGCGGTGACTCTG
SRRz _13	5'-TTGCAAACGCAAGGATTGCC		*SRRz* _36	5'-GGCAGACGATGATGCAGATA
SRRz _14	5'-TTTTTGTAAACGCACCGCCA		*SRRz* _37	5'-ACGGTAATGATTAACAGCCC
SRRz _15	5'-CACATCGTTGCGTCGATTAC		*SRRz* _38	5'-CTGGGCTTTGTAGGTAATGG
SRRz _16	5'-AATGAACCAGGCGATAATGG		*SRRz* _39	5'-TCAGTTCTCTGGCATTTTTG
SRRz _17	5'-TAAGCGAGATTGCTACTTAG		*SRRz* _40	5'-ATGTCAGTAATTGCCGCGTT
SRRz _18	5'-GCCGATAAACACGCTCGTTA		*SRRz* _41	5'-CTTCGTGTATTTTGCATCGA
SRRz _19	5'-CAATCGAGTCAGTACCGATG		*SRRz* _42	5'-TCAGCTTTAGCATCAGCTAA
SRRz _20	5'-GCGAAGCGTTTGATAAGCGA		*SRRz* _43	5'-AACATCATCACGCAGAGCAT
SRRz _21	5'-TTCTACTCCGGCTTTTTTAG		*SRRz* _44	5'-TTTGATGTGCAACCGACGAC
SRRz _22	5'-TCTGACGTCCGTTATCAGTT		*SRRz* _45	5'-TTCACGCACTGACTGACAGA
SRRz _23	5'-ATAGCTCTCCGCCTACAATG		*SRRz* _46	5'-GAGGGTGAAATAATCCCGTT

Supplementary Table 2. Probes for pR' FISH.

Name	Sequence	Comments
f-lambda-dnafish	AGAATCGACCATTTCTGCCATCACC	Forward primer for generating lambda DNA FISH probes
r-lambda-dnafish	GAGATTTCCGCTTTTGTGCTGGTTG	Reverse primer for generating lambda DNA FISH probes
f-attb-dnafish	GCGAGATTATCAAGGCGGGTA	Forward primer for generating *E. coli attB* DNA FISH probes
r-attb-dnafish	CGAATGGGATGAGCGCGATA	Reverse primer for generating *E. coli attB* DNA FISH probes
f-up-attb	TTAGCAGAAGTAAAGCGA	Forward primer upstream of *E. coli attB* for homology
r-up-attb	AACTACCCGCCTTGATAA	Reverse primer upstream of *E. coli attB* for homology
f-down-attb	TACAAATCGCATCGAACC	Forward primer downstream of *E. coli attB* for homology
r-down-attb	GGATGTGGATATCGTTTC	Reverse primer downstream of *E. coli attB* for homology
f-mid-dnab	ATTGCAGAAATCTCTCGCTCGCTGA	Forward primer inside *E. coli dnaB*
r-cmr	CAATGAAAGACGGTGAGCTGGTGAT	Reverse primer inside a Cm resistance cassette
r-h-dnab	GTTCGCTTTCACCACCGCAACCATT	Reverse primer downstream of *E. coli dnaB* for homology
r-down-dnab	ATGGCTGACGATATTCACCGGCTGA	Reverse primer downstream of *E. coli dnaB*, further downstream
upstreambor	GCACATCAAAGCAGTCTGTCAGTCAGTGCGTGAAGCCACCACCGCCTCCGGCGTGGATAATGCAGCCTCCCCCCGACTGGCAGACACCGCTGAACGGGATTATTTCACCCTCAGAGAGAGGCTGATCACTATGCAAAAACAACTGGAAGGAACCCAGAAGTATATTAATGAGCAGTGCAGATAGAGTTGCCCATATCGATG	Homology region upstream of the *bor* gene in the lambda genome used for recombination
downstreambor	AATGTTTTCACTTAATAGTATTGGTTGCGTAACAAAGTGCGGTCCTGCTGGCATTCTGGAGGGAAATACAACCGACAGATGTATGTAAGGCCAACGTGCTCAAATCTTCATACAGAAAGATTTGAAGTAATATTTTAACCGCTAGATGAAGAGCAAGCGCATGGAGCGACAAAATGAATAAAGAACAATCTGCTGATGAT	Homology region downstream of the *bor* gene in the lambda genome used for recombination
H	TTATTTTATGAATTAGGTAATTAAAGCAAACACTTATCAAGGAACACAAATGCAAGCGGCAACTGTTGTGATTAACCGCCGCGCTCTGCGACACAACCTGCAACGTCTTCGTGAACTGGCCCCTGCCAGTAAAATGGTTGCGGTGGTGAAAGCGAACGCTTATGGTCACGGTCTTCTTGAGACCGCGCGAACGCTCCCCGATGCTGACGCCTTTGGCGTAGCCCGTCTCGAAGAAGCTCTGCGACTGCGTGCGGGGGGAATCACCAAACCTGTACTGTTACTCGAAGGCTTTTTTGATGCCAGAGATCTGCCGACGATTTCTGCGCAACATTTTCATACCGCCGTGCATAACGAAGAACAGCTGGCT	Homology region downstream of the *E. coli dnaB* gene used for recombination

Supplementary Table 3. Primers and homology regions used in the study.

Supplementary References

1 Zeng, L. *et al.* Decision making at a subcellular level determines the outcome of bacteriophage infection. *Cell* **141**, 682-691, doi:10.1016/j.cell.2010.03.034 (2010).

2 Edgar, R. *et al.* Bacteriophage infection is targeted to cellular poles. *Molecular microbiology* **68**, 1107-1116, doi:10.1111/j.1365-2958.2008.06205.x (2008).

3 Tal, A., Arbel-Goren, R., Costantino, N., Court, D. L. & Stavans, J. Location of the unique integration site on an Escherichia coli chromosome by bacteriophage lambda DNA in vivo. *Proc Natl Acad Sci U S A* **111**, 7308-7312, doi:10.1073/pnas.1324066111 (2014).

4 Shao, Q., Hawkins, A. & Zeng, L. Phage DNA dynamics in cells with different fates. *Biophys J* **108**, 2048-2060, doi:10.1016/j.bpj.2015.03.027 (2015).

5 Mackay, D. & Bode, V. Events in lambda injection between phage adsorption and DNA entry. *Virology* **72** (1976).

6 Shao, Q. *et al.* Lysis-lysogeny coexistence: prophage integration during lytic development. *Microbiologyopen* **6**, doi:10.1002/mbo3.395 (2017).

7 Wang, X., Possoz, C. & Sherratt, D. J. Dancing around the divisome: asymmetric chromosome segregation in Escherichia coli. *Genes Dev* **19**, 2367-2377, doi:10.1101/gad.345305 (2005).

8 Kourilsky, P. Lysogenization by bacteriophage lambda-II. Identification of genes involved in the multiplicity dependent processes. *Biochimie* **56**, 1511-1515 (1974).

9 Trinh, J. T., Szekely, T., Shao, Q., Balazsi, G. & Zeng, L. Cell fate decisions emerge as phages cooperate or compete inside their host. *Nat Commun* **8**, 14341, doi:10.1038/ncomms14341 (2017).
