# Supplementary material for: Emerging heterogeneous compartments by viruses in single bacterial cells
Source: Nat Commun. 2020 Jul 30;11:3813. doi: 10.1038/s41467-020-17515-8 (PMC7393140; doi:10.1038/s41467-020-17515-8)
Supplement: Supplementary file 3 — Description of Additional Supplementary Files [file 41467_2020_17515_MOESM3_ESM.docx]

**Supplementary Movie Legends**

File Name: Supplementary Movie 1

Description: Example of uninfected reporter cell growth. Cells (LZ1557) grow on an M9M agarose pad.

File Name: Supplementary Movie 2

Description: Example of a lysogenic cell. Cell (LZ1557) infected by reporter phage (λLZ1576) undergoes lysogenic development.

File Name: Supplementary Movie 3

Description: Example of a lytic cell with one phactory. Cell (LZ1557) infected by reporter phage (λLZ1576) undergoes lytic development with one subcellular zone of DNA replication.

File Name: Supplementary Movie 4

Description: Example of a lytic cell with 2 phactories. Cell (LZ1557) infected by reporter phage (λLZ1576) undergoes lytic development with two subcellular zones of DNA replication.

File Name: Supplementary Movie 5

Description: Example of *attB* movement in a non-lytic cell. Cell (LZ1643) with *attB* reporter grows and divides.

File Name: Supplementary Movie 6

Description: Phage DNA pushes *attB* in a lytic cell. Cell (LZ1643) infected by reporter phage (λLZ1629) undergoes lytic development where *attB* is pushed to one side of the cell by phage DNA.

File Name: Supplementary Movie 7

Description: Phage DNA spreads *attB* in a lytic cell. Cell (LZ1643) infected by reporter phage (λLZ1629) undergoes lytic development where *attB* is spread to opposite cell poles by phage DNA.

File Name: Supplementary Movie 8

Description: Phage DNA squeezes *attB* in a lytic cell. Cell (LZ1643) infected by reporter phage (λLZ1629) undergoes lytic development where *attB* is squeezed between phage DNAs.
