# Supplementary material for: Emerging heterogeneous compartments by viruses in single bacterial cells
Source: Nat Commun. 2020 Jul 30;11:3813. doi: 10.1038/s41467-020-17515-8 (PMC7393140; doi:10.1038/s41467-020-17515-8)
Supplement: Supplementary file 13 — Source Data [file 41467_2020_17515_MOESM13_ESM.zip › SourceData/Source Data Information.docx]

**Source Data Information**

for

**Emerging Heterogeneous Compartments by Viruses in Single Bacterial Cells**

Jimmy T. Trinh, Qiuyan Shao, Jingwen Guan, and Lanying Zeng

livecellinf1.mat: This is the first live infection movie with the dnaB-FP reporter. It's used for Fig. 1, 4q, S1a-d, S2b-e, S3-a-d, S4, S5a, S7, and S9a,c.

livecellinf2.mat: This is the second live infection movie with the tet/lac reporters. It's used for Fig. 3, S5c, S9b,d, S10, and S11.

Induction.mat: This is the induction experiment in Fig. 2, S5b, S6, S8, and S15e.

dnafish.mat: This is the DNA FISH experiment used in Fig. 3a-d, g-k, p, S1f, S12, and S13.

rnafish1.mat: This is the RNA FISH experiment at 15 min with pR, pRE, and pR'. It's used for Fig. 4e-f, l-o, Fig. 5, S1e, and S14b-i.

rnafish2.mat: This is the RNA FISH experiment at multiple time points with PR. It's used for Fig. S14a.

riffishXmin.mat: This series of files is the RNA FISH experiments with rifampicin, what you can do is concatenate them into a single file like the others. It's used for Fig. S15a-d, f-k.

seqacontrol.xls: This is only file with cell counts with the seqA frequencies for Fig. S3a.

FigS2.xlsx: The raw data for Supplementary Fig. 2.
